# Local Coordination‐Dependent CO_2_ Reduction Activity of Bimetallic Cu─Al Catalysts for Selective Ethylene/Ethanol Electrosynthesis

**DOI:** 10.1002/anie.202520291

**Published:** 2025-11-10

**Authors:** Weihua Guo, Xingyu Wang, Yangbo Ma, Yun Song, Liang Chang, Haoran Wu, Geng Li, Zhihao Li, Yinger Xin, MingMing He, Jixun Zhang, Tao Yang, Minghui Zhu, Hanchen Shen, Shibo Xi, Xue Wang, Lin Gan, Qiu Jiang, Chuang Xia, Shenlong Zhao, Zhengxiao Guo, Ziyun Wang, Ben Zhong Tang, Ruquan Ye

**Affiliations:** ^1^ Department of Chemistry State Key Laboratory of Marine Environmental Health City University of Hong Kong Hong Kong 999077 P.R. China; ^2^ City University of Hong Kong Shenzhen Research Institute Shenzhen Guangdong 518057 P.R. China; ^3^ School of Chemical Sciences University of Auckland Auckland 1010 New Zealand; ^4^ Department of Chemistry The University of Hong Kong Hong Kong 999077 P.R. China; ^5^ Institute of Materials Research Tsinghua Shenzhen International Graduate School Tsinghua University Shenzhen 518055 P.R. China; ^6^ State Key Laboratory of Chemical Engineering East China University of Science and Technology Shanghai 200237 P.R. China; ^7^ Department of Materials Science and Engineering City University of Hong Kong Hong Kong 999077 P.R. China; ^8^ School of Science and Engineering Shenzhen Institute of Aggregate Science and Technology The Chinese University of Hong Kong Shenzhen (CUHK‐Shenzhen) Guangdong 518172 P.R. China; ^9^ Institute of Chemical and Engineering Sciences A*STAR Singapore 627833 Singapore; ^10^ School of Energy and Environment City University of Hong Kong Hong Kong 999077 P.R. China; ^11^ School of Materials and Energy University of Electronic Science and Technology of China Chengdu Sichuan 611731 P.R. China; ^12^ CAS Key Laboratory of Nanosystem and Hierarchical Fabrication CAS Center for Excellence in Nanoscience National Center for Nanoscience and Technology Beijing 100190 P.R. China

**Keywords:** Bimetallic catalysts, Carbon dioxide reduction reaction, Coordination‐dependent, Ethylene/Ethanol selectivity, Laser synthesis

## Abstract

The formation of bimetallic catalysts has been widely adopted to improve CO_2_ reduction selectivity. However, discrepancies in product distribution in the literature, even among catalysts with identical bimetal compositions, suggest the involvement of distinct reaction pathways. Here, we report that ethylene and ethanol selectivity are strongly influenced by the atomic coordination of metals. We prepared two model catalysts, namely interface‐CuAl (dominated by Cu/CuAlO_2_ interfaces) and doping‐CuAl (with Al doped into the Cu lattice). Both catalysts demonstrate excellent C_2+_ Faradaic efficiency (FE) of 65%–85%. However, interface‐CuAl primarily produces ethylene with an FE of 67.6%, seven‐fold higher than FE_ethanol_. Conversely, doping‐CuAl favors ethanol production, reaching a maximum FE_ethanol_ of 43.7%, four times higher than FE_ethylene_. Extended X‐ray absorption fine structure and in situ Fourier transform infrared spectrometry reveal distinct adsorption abilities of Cu and different intermediate coverages. Complementary theoretical calculation further elucidates the critical role of *CHCOH bifurcation. Specifically, favorable C–O cleavage at interface‐CuAl promotes ethylene production, whereas Cu–C scission at doping‐CuAl favors ethanol production. Beyond CO_2_ electroreduction, CuAl catalysts also demonstrate phase‐dependent nitrate reduction activity, underscoring the importance of atomic coordination in catalysis. This study provides fundamental insights into the structure‐selectivity relationship of bimetallic catalysts for selective chemical production.

## Graphical abstract

While bimetallic catalysts enhance C_2+_ yield, product selectivity remains elusive. We demonstrate that atomic coordination governs the bifurcation at the *CHCOH intermediate: Cu/CuAlO_2_ interface‐rich catalyst cleaves C–O to favor ethylene, whereas Al‐doped Cu catalyst breaks Cu–C to boost ethanol. This structure‐selectivity relationship, validated by spectroscopy and theory, establishes a design principle for selective CO_2_ electroreduction.

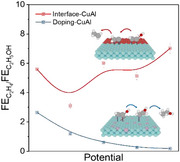

## Introduction

Renewable energy‐driven CO_2_ reduction reaction (CO_2_RR) can mitigate carbon emissions while producing value‐added chemicals. Cu‐based electrocatalysts have great potential for facilitating CO_2_ reduction to produce energy‐intensive fuels and chemicals such as ethylene (C_2_H_4_) and ethanol (C_2_H_5_OH).^[^ ^1^, ^2^, ^3^, ^4^ ^]^ Tremendous effort has been devoted to improving C_2+_ product selectivity by enhancing the binding energy of adsorbed CO (*CO) or increasing local CO_2_ concentrations.^[^ ^5^, ^6^, ^7^ ^]^ However, it remains challenging to improve the selectivity of a single C_2_ product that often shares similar reaction pathways.^[^ ^8^, ^9^, ^10^, ^11^ ^]^ Recent studies suggest the pathways toward C_2_H_4_ and C_2_H_5_OH share several common intermediates until a bifurcation step at the post‐C–C coupling stage of *CHCOH.^[^ ^8^, ^10^, ^12^, ^13^ ^]^ Specifically, selective C─O bond cleavage will favor the formation of C_2_H_4_, while further hydrogenation of *CHCOH will form C_2_H_5_OH. Thus, the relative stability of a selectivity‐determining intermediate (SDI) determines the bifurcation leading to C_2_H_4_ or C_2_H_5_OH.^[^ ^8^, ^14^ ^]^

Controlling the branching post‐C–C coupling is a key step towards C_2_H_4_ and C_2_H_5_OH selection. Introducing different metal atoms is a common strategy for modifying the adsorption of key intermediates on the catalyst surface, thus tuning the C_2+_ product selectivity.^[^ ^7^, ^15^, ^16^, ^17^, ^18^, ^19^ ^]^ For example, Li et al. demonstrated that Rh could control the oxygen binding strength of the Cu surface and favor C─O bond scission, which improved the selectivity of ethylene over ethanol.^[^ ^8^ ^]^ Peng et al. found that Cu_2_Mg intermetallic compound enhanced stabilization of the *CHCHOH intermediate, which boosted the formation of ethanol.^[^ ^20^ ^]^ The selectivity between Cu─C or the C─O bond cleavage can be used to predict the possibility of forming C_2_H_4_ and C_2_H_5_OH.^[^ ^9^, ^12^, ^21^ ^]^ Hence, the precise design of catalysts to tune intermediate adsorption strength can lead to different C_2_ routes and tailor the C_2_H_4_ and C_2_H_5_OH selectivity.

Despite significant successes in directing C_2_ product selectivity by forming alloys, the underlying mechanism of the alien metals remains elusive. For example, while some groups reported that CuAg alloys facilitate the adsorption of the key intermediates for ethanol production,^[^ ^22^, ^23^, ^24^ ^]^ others found that ethylene can dominate by boosting carbon monoxide spillover at the CuAg interfaces.^[^ ^25^, ^26^, ^27^ ^]^ The discrepancy among different literature warrants different reaction pathways in these alloys. Understanding the working mechanisms of alien metals will be essential for the rational design of copper‐based catalysts for CO_2_RR. Here, we report the evolution of atomic coordination of bimetal alloys in directing post‐C–C coupling selectivity toward C_2_H_4_ or C_2_H_5_OH. Using CuAl as model alloys, we found that different coordination in alloys leads to variations in the stabilization and activation of *CHCOH intermediates, dictating the cleavage or preservation of the C─O bond. Experimentally, the interface‐CuAl catalyst, which contains abundant CuO/CuAl_2_O_4_ interfaces, exhibits an outstanding ethylene Faradaic efficiency (FE) of 67.6 ± 4.3% and a partial current density of 283.6 mA cm^−2^. In comparison, doping‐CuAl, where the Al dopes into the lattice of Cu, is prone to produce ethanol with an FE of 43.7 ± 2.2% and a partial current density of 254.2 mA cm^−2^. The extended X‐ray absorption fine structure (EXAFS) and in situ attenuated total reflection Fourier transform infrared spectroscopy (ATR‐FTIR) reveal that interface‐CuAl with unsaturated copper sites shows stronger adsorption capacity, which elongates/weakens C─O bond of *CHCOH and stabilizes *CCH adsorption. In contrast, doping‐CuAl promotes C_2_H_5_OH formation by preserving the C─O bond due to the weaker adsorption capacity. Our studies highlight that the different coordination modes of Cu‐based alloys considerably influence the adsorption energy of key intermediates, thereby affecting the reaction pathway and product selectivity.

## Results and Discussion

### Catalyst Synthesis and Characterization

**Figure 1:**
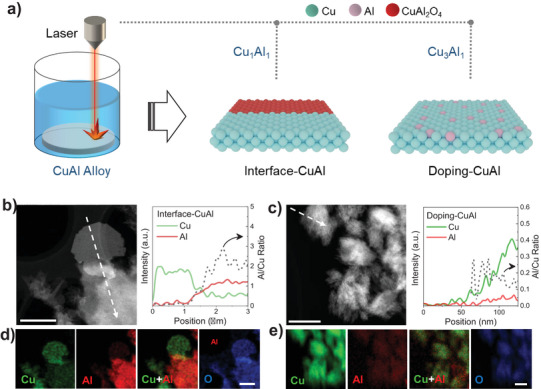
Morphology characterization. a) Schematic diagram of process in lasing Laser‐generated catalysts. b) and c) TEM and corresponding line scan intensity of interface‐CuAl and doping‐CuAl. d) and e) EDX mapping images of interface‐CuAl and doping‐CuAl. Scale bar: Scale bar: 1 µm in (b) and (d) and 100 nm in (c) and (e).

**Figure 2:**
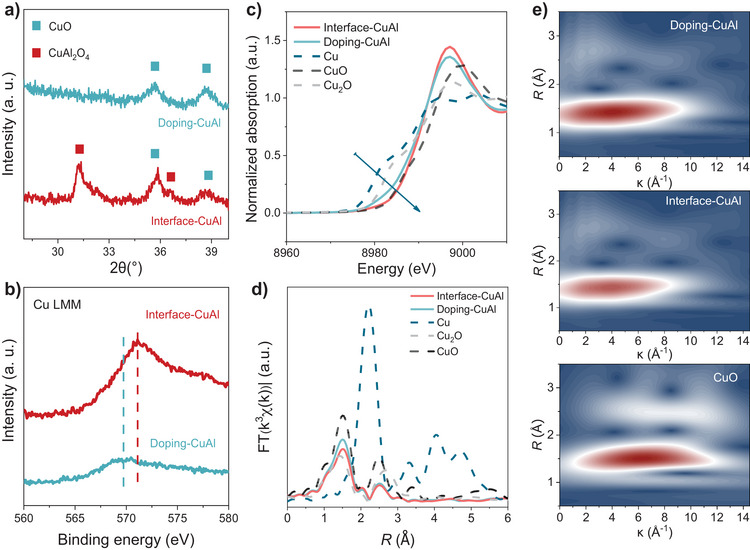
Structure characterization. a) XRD pattern and b) XPS pattern of interface‐CuAl and doping‐CuAl. c) Normalized XANES, d) Fourier transformed EXAFS, and e) WT–EXAFS Cu K‐edge spectra of interface‐CuAl, doping‐CuAl, C─CuO, and C─Cu.

The interface‐CuAl and doping‐CuAl NPs were prepared using a laser ablation process. As shown in Figure 1a, a CuAl target with different Cu:Al ratios was transiently heated by a pulsed laser to form a vapor or plasma state underneath water, which was then quenched by the cool water to the solid state.^[^ ^28^, ^29^, ^30^ ^]^ During this process, CuAl with an atomic ratio of 1:1 formed a hybrid with rich CuAlO_2_/CuO interfaces, signified as interface‐CuAl. Instead, using CuAl with a ratio of 3:1 as the target will produce Al‐doped CuO, termed as doping‐CuAl. High angular annular dark field scanning transmission electron microscopy (HAADF‐STEM) results and the corresponding linear scanning spectrogram of interface‐CuAl show that the elemental compositions significantly change across the boundary in interface‐CuAl, while doping‐CuAl has a relatively constant Cu:Al ratio along the linear scan (Figure 1b,c). Compositions analysis from inductively coupled plasma and energy‐dispersive spectroscopy shows that the Cu:Al ratio is roughly 7:3 and 9:1 for interface‐CuAl and doping‐CuAl, respectively (Table S1). Elemental mapping profiles further reveal the different elemental distributions on the interface‐CuAl and doping‐CuAl (Figure 1d,e). The elemental mapping profiles of interface‐CuAl show the phase‐separated CuO and CuAl_2_O_4_. In contrast, Cu, O, and Al elements were distributed evenly throughout the doping‐CuAl (Figures S1–S5). We further use X‐ray diffraction (XRD) to confirm the structure of doping‐CuAl and interface‐CuAl. The XRD patterns of both interface‐CuAl and doping‐CuAl catalysts (Figures 2a and S6) exhibit intense diffraction peaks assigned to the CuO phase. In addition to the CuO signals, the interface‐CuAl has additional peaks at 31.26° and 36.83°, attributed to the (220) and (311) facets of CuAl_2_O_4_, respectively.^[^ ^21^, ^31^ ^]^ However, doping‐CuAl only contains CuO peaks without the presence of AlO*_x_* signals.

We use high‐resolution X‐ray photoelectron spectroscopy (XPS) to understand the electronic structure of copper in the two catalysts. The Cu LMM peak position of interface‐CuAl lies at higher binding energy, which suggests the electron density of Cu in interface‐CuAl is lower than that in doping‐CuAl (Figures 2b and S7 and S8).^[^ ^6^, ^11^, ^31^, ^32^, ^33^ ^]^ The electronic structure is also investigated using X‐ray absorption spectroscopy at the Cu k‐edge. As the X‐ray absorption near‐edge structure (XANES) shows (Figure 2c), the absorption edges of interface‐CuAl and doping‐CuAl catalysts lie between the spectrum features of commercial standard C─CuO and C─Cu_2_O, indicating the existence of the complex state between Cu^+^ and Cu^2+^ for interface‐CuAl and doping‐CuAl electrocatalysts. The absorption edge of interface‐CuAl is located at higher energy, which suggests that it has a higher oxidation state than doping‐CuAl, consistent with the XPS analyzes.^[^ ^31^, ^34^, ^35^, ^36^ ^]^ These results suggest that the different Al structural statuses can lead to variations in Cu electronic structures.

The Fourier‐transformed EXAFS spectrum was further applied to investigate the coordination environment of the Cu species in the samples. As shown in Figure 2d, the first‐shell intensity of interface‐CuAl, doping‐CuAl, and C─CuO are very similar, confirming that the Cu─O bonds are dominant in all these samples, matching well with the EXAFS fitting results (Figures S9 and S10; Table S2). The wavelet transform was used to visualize the local coordination environments (Figure 2e). The maximum intensity for interface‐CuAl in the k space for the first coordination shell (Cu─O) is smaller than that of doping‐CuAl and C─CuO, which suggests the unsaturated copper in interface‐CuAl structure.^[^ ^9^, ^30^, ^37^ ^]^ The unsaturation structure of interface‐CuAl would lead to the upward of the d‐band center of Cu, which lead to stronger absorption that facilitates the Cu─C bonding.^[^ ^9^, ^12^ ^]^

### Electrochemical CO_2_ Reduction Reaction

**Figure 3:**
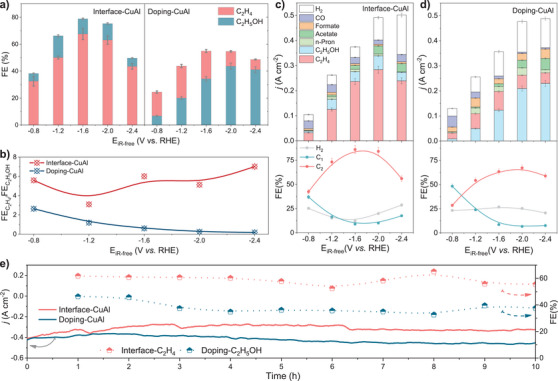
CO_2_ electroreduction performance. a) FE value of C_2_H_4_ and C_2_H_5_OH on interface‐CuAl and doping‐CuAl at different applied potentials (without iR‐correction). b) Ratio value of FE C_2_H_4_ / C_2_H_5_OH on interface‐CuAl and doping‐CuAl. c) and d) Current density (top) and FE values (bottom) of each CO_2_RR product and H_2_ on interface‐CuAl and doping‐CuAl at different applied potentials. e) Stability measurement on interface‐CuAl and doping‐CuAl. The stability tests were performed at a potential of ‐2.0 V versus RHE. Error bars represent the standard deviation of three independent measurements.

The CO_2_RR measurements of different catalysts were tested in flow cells (Figures S11–S18 and Methods). The interface‐CuAl shows a higher FE of C_2_H_4_ in the full potential range, reaching a peak value of 67.6% ± 4.3%. Doping‐CuAl shows higher ethanol production with a maximum FE of 43.7% ± 2.2% (Figure 3a). The ethylene to ethanol FE ratio is 5.6 at ‐0.8 V and maintains above 4.0 for interface‐CuAl (Figure 3b). This ratio is higher than most literature report for C_2_H_4_ electrosynthesis (red shaded area).^[^ ^8^ ^]^ In comparison, the ethylene to ethanol ratio of doping‐CuAl is 2.6 at ‐0.8 V and decreases to 0.2 at higher overpotentials; this value is lower than other literature data for C_2_H_5_OH production,^[^ ^38^ ^]^ underscoring the high selectivity of C_2_H_5_OH. The CO_2_RR current densities generally increase with applied potentials (Figure 3c,d); the partial current density of C_2+_ products in interface‐CuAl reaches 378.5 mA cm^−2^ with a corresponding FE_C2+_ of 86.0 ± 4.3% at −1.6 V. Both bimetal catalysts exhibit high selectivity of C_2+_ products: The FE_C2+_ of the doping‐CuAl reaches its peak value of 67.3 ± 3.4% at −2.0 V, alongside a partial current density of 322.7 mA cm^−2^. They also exhibit good durability and work steadily under a high current density of ∼400 mA cm^−2^ for 10 h with an average C_2_H_4_ selectivity of 60% and C_2_H_5_OH selectivity of 40%, respectively (Figures 3e and S19–S22). Although both doping‐CuAl and interface‐CuAl underwent varying degrees of reconstruction during the reduction process, the unique coordination environment of interface‐CuAl enabled it to retain a higher proportion of oxidized copper species. This structural preservation is crucial for maintaining high ethylene selectivity, as further corroborated by its stability performance (Figures 3e and S19).

### Mechanistic Studies from In Situ Spectroscopy

**Figure 4:**
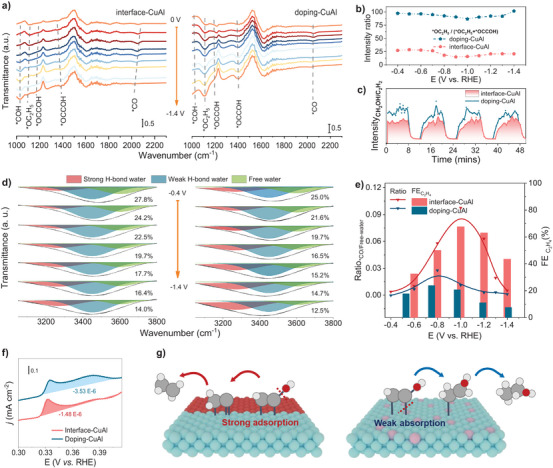
Mechanism investigation. a) In situ ATR‐FTIR of CO_2_RR from 0 V to −1.4 V for interface‐CuAl (left) and doping‐CuAl (right). b) I_*OC2H5_ /I_*OC2H5_ + I_*OCCOH_ ratio of interface‐CuAl and doping‐CuAl. c) Online DEMS of interface‐CuAl and doping‐CuAl for ratio of CH_2_OH /C_2_H_2_ (ethanol: ─CH_2_OH; C_2_H_4_: ─C_2_H_2_). d) Potential‐dependent fitted bands of interfacial water at 3850–3050 cm^−1^ for interface‐CuAl (left) and doping‐CuAl (right). The percentage indicates the portion of free water. e) The relationship between *CO/Free‐water with FE of C_2_H_4_ on interface‐CuAl and doping‐CuAl; the FE data is taken from flow cell with iR compensation. f) OH^‒^ absorption test at interface‐CuAl and doping‐CuAl. g) Schematic illustration of catalytic process on interface‐CuAl and doping‐CuAl; cyan‐blue: copper, red: aluminum acid copper, pink: aluminum, gray: carbon, white: hydrogen, light red: oxygen.

Our experimental data suggest that structural variations in CuAl catalysts can affect the catalytic activities. To understand the mechanistic origin, we first performed in situ ATR‐FTIR to probe the dynamic evolution of surface adsorptions during CO_2_RR.^[^ ^29^ ^]^ For ATR‐FTIR in transmission mode, the downward band in the spectra indicates the formation of intermediates, while the upward band refers to the consumption/desorption of surface species.^[^ ^39^ ^]^ As shown in Figures 4a and S23 and Table S4, two peaks at ∼1034 and ∼1400 cm^−1^ associated with *COH and *OCCOH,^[^ ^22^, ^27^, ^40^, ^41^ ^]^ respectively, are observed for the interface‐CuAl and doping‐CuAl, which are important intermediates for C_2+_ products. The peaks at ∼1130 cm^−1^ is related to the *OC_2_H_5_,^[^ ^6^, ^22^ ^]^ which is an important intermediate for ethanol. The intensity ratio of *OC_2_H_5_ to *OC_2_H_5_ +*OCCOH was calculated to study the ethanol conversion capacity of the interface‐CuAl and doping‐CuAl (Figure 4b). According to the result, the higher ratio of doping‐CuAl indicates that more key intermediates of *OCCOH have been readily transferred to *OC_2_H_5_, which favors the production of ethanol on doping‐CuAl than on interface‐CuAl. We further investigate the time‐dependent evolution of in situ ATR‐FTIR (Figure S24). Both samples show the gradual conversion of *CO into *OCCOH. However, the intensity of the characteristic absorption peak of *OC_2_H_5_ at approximately 1130 cm^−1^ for the doping‐CuAl catalyst exhibits a continuous and more pronounced increase than that of the interface‐CuAl catalyst with the progression of the reaction time. This observation strongly indicates that the doping‐CuAl catalyst possesses a higher propensity for the production of ethanol during the reaction process, as evidenced by the enhanced formation of the *OC_2_H_5_ species, in contrast to the interface‐CuAl catalyst. Moreover, the online differential electrochemical mass spectrometry (DEMS) was used to detect product distribution (Figure 4c). The ratio of ─CH_2_OH/─C_2_H_2_ on doping‐CuAl is higher than that on interface‐CuAl at all potential ranges, indicating the better ethanol production of doping‐CuAl.

To conduct a qualitative evaluation of the impact of the interfacial water microenvironment on CO_2_RR under high current densities, the peaks of interfacial water absorption bands near 3600 cm^−1^ were deconvoluted into three components. Specifically, the peaks at 3600, 3450, and 3270 cm^−1^ were attributed to free water, two‐coordinated hydrogen‐bonded water (weakly hydrogen‐bonded 2‐HB water), and four‐coordinated hydrogen‐bonded water (strongly hydrogen‐bonded 4‐HB water), respectively. The quantitatively normalized peak area ratios are illustrated in Figure 4d. According to molecular interaction theory, enhanced hydrogen‐bonding weakens the O─H vibrational energy, resulting in an ascending order of water dissociation energies: free H_2_O < 2‐HB H_2_O < 4‐HB H_2_O. Notably, the interface‐CuAl catalyst and doping‐CuAl have a moderate molar fraction of free H_2_O. This suggests the interface‐CuAl and doping‐CuAl surface is rich in free H_2_O, providing abundant active protons for *CO deep hydrogenation to C_2_H_4_ and ethanol.^[^ ^11^, ^42^, ^43^, ^44^ ^]^ We compared FE_C2H4_ in interface‐CuAl and doping‐CuAl, which exhibits a similar trend with the *CO/Free‐water populations, suggesting that the ratio of *CO/Free‐water coverage may be key for the formation of C_2_H_4_ (Figure 4e). The ATR‐FTIR results have provided experimental evidence that different coordination modes change the intermediate coverages in the post‐C–C coupling, which impacts the product selectivity.

We then use cyclic voltammetry (CV) to verify the OH^‒^ adsorption (OH_ads_) features, which have been reported to correlate with the ethylene and ethanol selectivity.^[^ ^45^, ^46^, ^47^ ^]^ It has been observed that the more favorable *OH coverage can promote the ethanol formation pathway.^[^ ^6^, ^45^ ^]^ Figure 4f demonstrates the pronounced OH_ads_ peaks associated with Cu (100) facets on the catalysts, where the OH_ads_ peaks are located at ∼0.335 V for interface‐CuAl and doping‐CuAl, respectively.^[^ ^6^, ^12^, ^29^, ^48^ ^]^ The adsorption capacity of OH_ads_ peaks in doping‐CuAl (3.53 × 10^−6^ C) is 2.4 times higher than that in interface‐CuAl (1.48 × 10^−6^ C), which can facilitate ethanol generation. These results indicate that the different coordination modes would change the surface interaction with adsorbates, thereby resulting in different selectivity.

Based on the above result, we speculate that after the three proton‐coupled electron transfer steps of the *OCCOH intermediate to form *CHCOH species,^[^ ^8^, ^9^ ^]^ the different surface adsorption capacities of CuAl catalysts determine the ethylene or ethanol production. Specifically, we conducted a comprehensive analysis of the reaction energies associated with the hydrogenation of the *CHCOH intermediate, which can proceed via two distinct pathways leading to *CHCHOH (the precursor for ethanol formation) and *CCH (the precursor for ethylene formation), as illustrated in Figures 4g and S26. This particular hydrogenation step plays a pivotal role in determining the selectivity between ethanol and ethylene production. The interface‐CuAl has a stronger adsorption capacity. Thus, its C_2_ pathway proceeds via C─O bond scission of *CHCOH, transforming into *C–*CH and eventually forming the C_2_H_4_ (Figure 4g, left). In contrast, doping‐CuAl has a weaker adsorption capacity, which favors the Cu─C bond cleavage, followed by hydrogenation of the α‐carbon in *CH_2_COH to form *CH_3_CHO, consequently affording C_2_H_5_OH (Figure 4g, right).

### Mechanistic Insights from Theoretical Calculations

**Figure 5:**
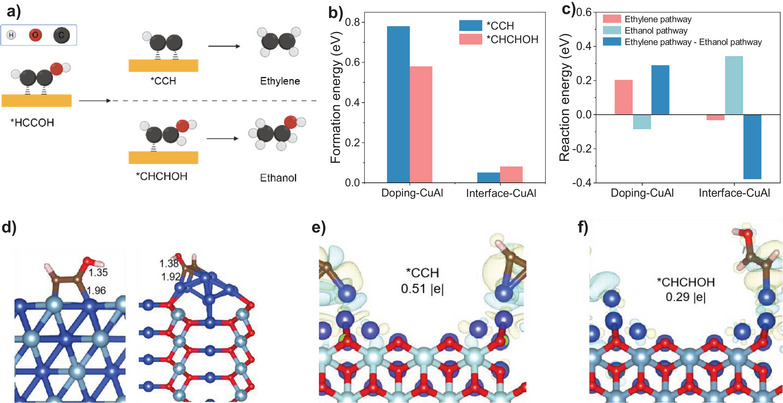
DFT calculations of post‐C–C coupling steps on different coordination modes catalysts. a) The key intermediates of ethylene pathway and ethanol pathway. b) Formation energies of the *CCH and *CHCHOH on doping‐CuAl and interface‐CuAl. c) Reaction energies of the ethylene pathway (*CHCOH to *CCH) and the ethanol pathway (*CHCOH to *CHCHOH) on doping‐CuAl and interface‐CuAl. d) The length of C─OH, C─Cu on doping‐CuAl, and interface‐CuAl, the color scheme for atoms is as follows: dark blue for Cu, light blue for Al, red for O, gold for C, and white for H. e) and f) Electron density difference plots for interface‐CuAl with adsorbed *CCH and *CHCHOH. Yellow contours represent charge accumulations, and blue contours denote charge depressions, dark blue for Cu, light blue for Al, red for O, gold for C, and white for H.

We further use density functional theory calculations to understand the reaction energies of different surface adsorptions. Figure 5a illustrates two branching reaction pathways for the conversion of *CHCOH.^[^ ^13^, ^38^, ^40^, ^41^, ^49^ ^]^ The upper pathway proceeds through C─O bond cleavage to form *CCH intermediate, eventually generating ethylene (C_2_H_4_). The alternative pathway goes through *CHCHOH intermediate to produce ethanol (C_2_H_5_OH). To investigate the selectivity of the preferred reaction pathway, we calculated the formation energies of key intermediates *CCH and *CHCHOH on surfaces (Figures 5b and S26–S32). We chose two types of surfaces: doping‐CuAl (Al‐doped Cu) and interface‐CuAl (Cu/CuAlO_2_) interfaces. On the doping‐CuAl surface, *CHCHOH exhibits lower formation energy than the *CCH, suggesting it is more prone to form *CHCHOH, leading to ethanol formation. However, on the interface‐CuAl surface, the formation energy of *CCH is lower than that of *CHCHOH, implying that *CCH formation is more favorable, thus primarily forming ethylene. Our calculations highlight the significant role of bimetal phases in directing the reaction pathway toward the desired products.

Since *CHCOH is a critical intermediate at the branching point between the ethylene and ethanol pathways, we further calculated the free energy change from *CHCOH to *CCH (ethylene pathway) or to *CHCHOH (ethanol pathway), as shown in Figure 5c. On the interface‐CuAl surface, compared to the doping‐CuAl surface, the reaction energy for the ethylene pathway decreased from 0.20 to ‐0.04 eV, while the reaction energy for the ethanol pathway increased from ‐0.09 eV to 0.34 eV. To more accurately compare selectivity, we subtracted the free energy change of the ethanol pathway from that of the ethylene pathway on the same surface. On the doping‐CuAl surface, the ethylene pathway exhibits a higher reaction energy, indicating that ethanol is the predominant product. Conversely, on the interface‐CuAl surface, the ethanol pathway shows a higher reaction energy, suggesting that ethylene is the favored product. These findings align with experimental observations.

To elucidate the origin of this selectivity, we analyzed the configurations of the key intermediate *CHCOH on both interface‐CuAl and doping‐CuAl surfaces, as illustrated in Figure 5d. On the interface‐CuAl surface, the Cu─C bond length in *CHCOH is measured to be 1.92 Å, while the C─O bond length is 1.38 Å. In contrast, on the doping‐CuAl surface, the Cu─C bond length extends to 1.96 Å, with a corresponding C─O bond length of 1.35 Å. These structural variations have important implications for the reaction mechanism. The longer C─O bond (1.38 Å versus 1.35 Å) on the interface‐CuAl surface suggests a weakened C─O interaction, facilitating easier bond cleavage. In contrast, the shorter Cu─C bond (1.92 Å versus 1.96 Å) on the interface‐CuAl indicates a stronger metal‐carbon interaction, which is more conducive to the formation of the *CCH intermediate. This configuration analysis provides a molecular‐level explanation for the observed selectivity. The interface‐CuAl interface significantly influences the geometry of the critical *CHCOH intermediate, altering bond lengths in a way that energetically favors the ethylene pathway over the ethanol pathway. These structural insights corroborate our earlier energetic calculations.

To elucidate the slab's influence on key intermediates, we generated electron density difference plots for interface‐CuAl with adsorbed *CCH and *CHCHOH (Figure 5e). The plot reveals that the interface‐CuAl layer consistently acts as an electron donor (blue regions). In contrast, *CCH exhibits uniform electron accumulation (yellow regions), while *CHCHOH displays a more complex charge redistribution pattern with both electron gain and loss. We further quantified this charge transfer using Bader charge analysis. The results reveal that *CCH and *CHCHOH on the interface‐CuAl surface transfer 0.51 and 0.29 |e|, respectively. The larger charge transfer for *CCH on the interface‐CuAl surface indicates a stronger interaction between the adsorbate and the slab. This stronger interaction leads to lower adsorption energy, making *CCH formation more favorable. These findings support the hypothesis that the ethylene pathway is the dominant reaction route at the interfaces.

### Impact of Different Coordination Modes on Other Reaction

The different coordination modes might also benefit other multielectron electrochemical reactions.^[^ ^50^ ^]^ As a proof‐of‐concept demonstration, the electrocatalytic nitrate reduction reaction (NITRR) activity of catalysts was evaluated using the two catalysts in an H‐type cell (Figures S33–S42). Figure S40a,b show the performance of the interface‐CuAl and doping‐CuAl catalysts in different concentrations of KNO_3_. Both doping‐CuAl and interface‐CuAl reach >80% FE_NH3_ in the window from −0.4 to −1.0 V, implying the effectiveness of introducing alien metals in improving the catalytic activities. However, the doping‐CuAl generally has better FE performance than the interface catalyst, which can maintain a higher FE_NH3_ with lower KNO_3_ concentration at lower overpotentials. The comparison is clearer in terms of the ammonia yield rate; the NH_3_ yield rate of doping‐CuAl reaches 67 mg^−1^ h^−1^ mg_catalyst_^−1^ at −1.0 V, while that of interface‐CuAl is 47.98 mg^−1^ h^−1^ mg_catalyst_^−1^ at −1.0 V (Figure S40b).

We further used in situ ATR‐FTIR to track intermediates adsorbed on the surface of two catalysts. In Figure S41, two prominent absorption bands appear in the spectra of doping‐CuAl and interfaces‐CuAl catalysts: The downward band at 1236 cm^−1^ is attributed to N–O antisymmetric stretching vibration of *NO_2_, indicating NO_2_^−^ formation from NO_3_^−^ reduction; another intermediate observed around 1110 cm^−1^is ascribed to N─O stretching vibration of hydroxylamine (*NH_2_OH), which is a key intermediate for NH_3_ formation.^[^ ^51^ ^]^ With the negative shift of the working potential, the intensity ratio of *NH_2_OH relative to the sum area of *NO_2_ and *NH_2_OH on the doping‐CuAl is higher than that on the interface‐CuAl throughout the wide potential ranges, indicating that the doping‐CuAl can convert *NO_2_ faster than interface‐CuAl, facilitating the formation of final product NH_3_ (Figure S41a,b). This phenomenon is also consistent with results of electrocatalytic performance (Figure S40a,b). It has been reported that the relatively strong adsorption for the intermediate product nitrite can cause poisoning and thus reduce ammonia production.^[^ ^52^, ^53^, ^54^ ^]^ The interface‐CuAl, which has a stronger absorption ability than doping‐CuAl, could exacerbate the poisoning of the intermediate adsorbate, thereby reducing ammonia production.

## Discussion

Controlling the post‐C–C coupling pathway is essential for achieving high selectivity in the electrocatalytic CO_2_ reduction to C_2_ products. We demonstrate that tailoring the coordination environment of copper atoms effectively modulates the surface adsorption properties. Combined theoretical and experimental analyses establish adsorption strength as a general descriptor for predicting the bifurcation of the *CHCOH intermediate: strong adsorption at interface‐rich CuAl sites promotes C─O cleavage toward C_2_H_4_, whereas weak adsorption on doping‐type CuAl favors C─O retention and ethanol formation. This coordination‐driven strategy extends beyond CuAl, as evidenced by CuAg systems. We anticipate that similar principles apply to other bimetallic systems (e.g., CuAu, CuMg), where local coordination engineering—via doping, alloying, or interface design—can selectively steer the distribution of C_2_ products. By moving beyond simple alloying toward the deliberate creation of bi‐phase or even multiphase architectures, where isolated doping sites, intermetallic compounds, and well‐defined interfaces coexist in a single catalyst, it becomes possible to decouple and independently optimize distinct reaction steps. This opens a promising pathway not only for steering C–C coupling and C─O bond scission in CO_2_ reduction but also for managing multi‐electron reaction pathways across a broader range of electrocatalytic processes.

## Author Contributions

R.Y. conceived and designed the research. R.Y., Z.W., B.Z.T., and Z.X.G. supervised the research. W.G. carried out most of the experiments and X.W. performed the calculations. Y. M., H.W., M.Z., and Z.X. performed the in situ Fourier transform infrared studies. S.X. performed the X‐ray absorption spectroscopy experiment and analysis. Y.M., Y.S., G.L., Z.L., L.H., Y.X. M.H., J.Z. H.S., and X.W. conducted part of the experiments. R.Y. and W.G. analyzed the data and wrote the manuscript with input from the other authors.

## Conflict of Interests

The authors declare no conflict of interest.

## Supporting information



Supporting Information

## Data Availability

The data that support the findings of this study are available from the corresponding author upon reasonable request.

## References

[1] K. U. Hansen , F. Jiao , Nat. Energy 2021, 6, 1005–1006, 10.1038/s41560-021-00930-6.

[2] R. Buonsanti , Nat. Catal. 2021, 4, 736–737, 10.1038/s41929-021-00674-2.

[3] Y. Yao , T. Shi , W. Chen , J. Wu , Y. Fan , Y. Liu , L. Cao , Z. Chen , Nat. Commun. 2024, 15, 1257, 10.1038/s41467-024-45704-2. 38341442 PMC10858863

[4] H. Wu , L. Huang , J. Timoshenko , K. Qi , W. Wang , J. Liu , Y. Zhang , S. Yang , E. Petit , V. Flaud , J. Li , C. Salameh , P. Miele , L. Lajaunie , B. Roldán Cuenya , D. Rao , D. Voiry , Nat. Energy 2024, 9, 422–433, 10.1038/s41560-024-01461-6.

[5] A. Vasileff , C. Xu , Y. Jiao , Y. Zheng , S.‐Z. Qiao , Chem 2018, 4, 1809–1831, 10.1016/j.chempr.2018.05.001.

[6] L. Zhang , J. Feng , L. Wu , X. Ma , X. Song , S. Jia , X. Tan , X. Jin , Q. Zhu , X. Kang , J. Ma , Q. Qian , L. Zheng , X. Sun , B. Han , J. Am. Chem. Soc. 2023, 145, 21945–21954, 10.1021/jacs.3c06697. 37751566

[7] T. Zhang , B. Yuan , W. Wang , J. He , X. Xiang , Angew. Chem. Int. Ed. 2023, 62, e202302096. 10.1002/anie.202302096 37026583

[8] Z. Li , P. Wang , X. Lyu , V. K. R. Kondapalli , S. Xiang , J. D. Jimenez , L. Ma , T. Ito , T. Zhang , J. Raj , Y. Fang , Y. Bai , J. Li , A. Serov , V. Shanov , A. I. Frenkel , S. D. Senanayake , S. Yang , T. P. Senftle , J. Wu , Nat. Chem. Eng. 2024, 1, 159–169, 10.1038/s44286-023-00018-w.

[9] J. Jiao , X. Kang , J. Yang , S. Jia , Y. Peng , S. Liu , C. Chen , X. Xing , M. He , H. Wu , B. Han , J. Am. Chem. Soc. 2024, 146, 15917–15925, 10.1021/jacs.4c02607. 38805725

[10] W. Deng , P. Zhang , Y. Qiao , G. Kastlunger , N. Govindarajan , A. Xu , I. Chorkendorff , B. Seger , J. Gong , Nat. Commun. 2024, 15, 892, 10.1038/s41467-024-45230-1. 38291057 PMC10828390

[11] Y. Lin , T. Wang , L. Zhang , G. Zhang , L. Li , Q. Chang , Z. Pang , H. Gao , K. Huang , P. Zhang , Z. J. Zhao , C. Pei , J. Gong , Nat. Commun. 2023, 14, 3575, 10.1038/s41467-023-39351-2. 37328481 PMC10275897

[12] Z. Liu , L. Song , X. Lv , M. Liu , Q. Wen , L. Qian , H. Wang , M. Wang , Q. Han , G. Zheng , J. Am. Chem. Soc. 2024, 146, 14260–14266, 10.1021/jacs.4c03830. 38714344

[13] Y. Liang , F. Li , R. K. Miao , S. Hu , W. Ni , S. Zhang , Y. Liu , Y. Bai , H. Wan , P. Ou , X.‐Y. Li , N. Wang , S. Park , F. Li , J. Zeng , D. Sinton , E. H. Sargent , Nat. Synth. 2024, 3, 1104–1112, 10.1038/s44160-024-00568-8.

[14] J. Liu , B. Zhang , D. Chen , O. Peng , H. Ma , S. Xi , C. Wu , Q. Hu , K. Zhang , J. Feng , K. P. Loh , Angew. Chem. Int. Ed. 2024, 63, e202412266. 10.1002/anie.202412266 39158126

[15] T.‐T. Zhuang , Z.‐Q. Liang , A. Seifitokaldani , Y. Li , P. De Luna , T. Burdyny , F. Che , F. Meng , Y. Min , R. Quintero‐Bermudez , C. T. Dinh , Y. Pang , M. Zhong , B. Zhang , J. Li , P.‐N. Chen , X.‐L. Zheng , H. Liang , W.‐N. Ge , B.‐J. Ye , D. Sinton , S.‐H. Yu , E. H. Sargent , Nat. Catal. 2018, 1, 421–428, 10.1038/s41929-018-0084-7.

[16] C. Zhan , F. Dattila , C. Rettenmaier , A. Herzog , M. Herran , T. Wagner , F. Scholten , A. Bergmann , N. López , B. R. Cuenya , Nat. Energy 2024, 9, 1485–1496, 10.1038/s41560-024-01633-4. 39713047 PMC11659170

[17] Y. Zhang , Y. Chen , X. Wang , Y. Feng , Z. Dai , M. Cheng , G. Zhang , Nat. Commun. 2024, 15, 5172, 10.1038/s41467-024-49247-4. 38890306 PMC11189494

[18] L. L. Zhuo , P. Chen , K. Zheng , X. W. Zhang , J. X. Wu , D. Y. Lin , S. Y. Liu , Z. S. Wang , J. Y. Liu , D. D. Zhou , J. P. Zhang , Angew. Chem. Int. Ed. 2022, 61, e202204967, 10.1002/anie.202204967. 35510692

[19] Z.‐T. Yan , S. Tao , J. Wang , X.‐L. Lu , T.‐B. Lu , Adv. Mater. 2024, 36, 2411942, 10.1002/adma.202411942. 39340286

[20] C. Peng , J. Ma , G. Luo , S. Yan , J. Zhang , Y. Chen , N. Chen , Z. Wang , W. Wei , T. K. Sham , Y. Zheng , M. Kuang , G. Zheng , Angew. Chem. Int. Ed. 2024, 63, e202316907, 10.1002/anie.202316907. 38436539

[21] X. Wang , Y. Jiang , K. Mao , W. Gong , D. Duan , J. Ma , Y. Zhong , J. Li , H. Liu , R. Long , Y. Xiong , J. Am. Chem. Soc. 2022, 144, 22759–22766, 10.1021/jacs.2c11109. 36453117

[22] P. Wang , H. Yang , C. Tang , Y. Wu , Y. Zheng , T. Cheng , K. Davey , X. Huang , S. Z. Qiao , Nat. Commun. 2022, 13, 3754, 10.1038/s41467-022-31427-9. 35768462 PMC9243136

[23] X. Lv , L. Shang , S. Zhou , S. Li , Y. Wang , Z. Wang , T. K. Sham , C. Peng , G. Zheng , Adv. Energy Mater. 2020, 10, 2001987, 10.1002/aenm.202001987.

[24] A. Shayesteh Zeraati , F. Li , T. Alkayyali , R. Dorakhan , E. Shirzadi , F. Arabyarmohammadi , C. P. O'Brien , C. M. Gabardo , J. Kong , A. Ozden , M. Zargartalebi , Y. Zhao , L. Fan , P. Papangelakis , D. Kim , S. Park , R. K. Miao , J. P. Edwards , D. Young , A. H. Ip , E. H. Sargent , D. Sinton , Nat. Synth. 2024, 4, 75–83, 10.1038/s44160-024-00662-x.

[25] Y. Ma , J. Yu , M. Sun , B. Chen , X. Zhou , C. Ye , Z. Guan , W. Guo , G. Wang , S. Lu , D. Xia , Y. Wang , Z. He , L. Zheng , Q. Yun , L. Wang , J. Zhou , P. Lu , J. Yin , Y. Zhao , Z. Luo , L. Zhai , L. Liao , Z. Zhu , R. Ye , Y. Chen , Y. Lu , S. Xi , B. Huang , C. S. Lee , Z. Fan , Adv. Mater. 2022, 34, e2110607, 10.1002/adma.202110607. 35275439

[26] J. Huang , M. Mensi , E. Oveisi , V. Mantella , R. Buonsanti , J. Am. Chem. Soc. 2019, 141, 2490–2499, 10.1021/jacs.8b12381. 30657662

[27] S. Wang , F. Li , J. Zhao , Y. Zeng , Y. Li , Z. Y. Lin , T. J. Lee , S. Liu , X. Ren , W. Wang , Y. Chen , S. F. Hung , Y. R. Lu , Y. Cui , X. Yang , X. Li , Y. Huang , B. Liu , Nat. Commun. 2024, 15, 10247, 10.1038/s41467-024-54636-w. 39592645 PMC11599749

[28] Z. Li , J.‐Y. Fu , Y. Feng , C.‐K. Dong , H. Liu , X.‐W. Du , Nat. Catal. 2019, 2, 1107–1114, 10.1038/s41929-019-0365-9.

[29] W. Guo , S. Zhang , J. Zhang , H. Wu , Y. Ma , Y. Song , L. Cheng , L. Chang , G. Li , Y. Liu , G. Wei , L. Gan , M. Zhu , S. Xi , X. Wang , B. I. Yakobson , B. Z. Tang , R. Ye , Nat. Commun. 2023, 14, 7383, 10.1038/s41467-023-43303-1. 37968299 PMC10651938

[30] W. Guo , Y. Zhang , J. Su , Y. Song , L. Huang , L. Cheng , X. Cao , Y. Dou , Y. Ma , C. Ma , H. Zhu , T. Zheng , Z. Wang , H. Li , Z. Fan , Q. Liu , Z. Zeng , J. Dong , C. Xia , B. Z. Tang , R. Ye , Small 2022, 18, e2201311. 35561067 10.1002/smll.202201311

[31] S. Sultan , H. Lee , S. Park , M. M. Kim , A. Yoon , H. Choi , T.‐H. Kong , Y.‐J. Koe , H.‐S. Oh , Z. Lee , H. Kim , W. Kim , Y. Kwon , Energy & Environ. Sci. 2022, 15, 2397–2409, 10.1039/D1EE03861C.

[32] Y. Zang , T. Liu , P. Wei , H. Li , Q. Wang , G. Wang , X. Bao , Angew. Chem. Int. Ed. 2022, 61, e202209629, 10.1002/anie.202209629. 35909076

[33] G. Li , L. Huang , C. Wei , H. Shen , Y. Liu , Q. Zhang , J. Su , Y. Song , W. Guo , X. Cao , B. Z. Tang , M. Robert , R. Ye , Angew. Chem. Int. Ed. 2024, 63, e202400414, 10.1002/anie.202400414. 38348904

[34] C. Tebar‐Soler , V. Martin‐Diaconescu , L. Simonelli , A. Missyul , V. Perez‐Dieste , I. J. Villar‐Garcia , J. B. Brubach , P. Roy , M. L. Haro , J. J. Calvino , P. Concepcion , A. Corma , Nat. Mater. 2023, 22, 762–768, 10.1038/s41563-023-01540-1. 37142737

[35] W. Zhang , C. Huang , J. Zhu , Q. Zhou , R. Yu , Y. Wang , P. An , J. Zhang , M. Qiu , L. Zhou , L. Mai , Z. Yi , Y. Yu , Angew. Chem. Int. Ed. 2022, 61, e202112116, 10.1002/anie.202112116. 34704659

[36] G. Shi , Y. Xie , L. Du , X. Fu , X. Chen , W. Xie , T. B. Lu , M. Yuan , M. Wang , Angew. Chem. Int. Ed. 2022, 61, e202203569, 10.1002/anie.202203569. 35301781

[37] J. Li , M. Li , N. An , S. Zhang , Q. Song , Y. Yang , X. Liu , Proc. Natl. Acad. Sci. USA 2021, 118, e2105628118, 10.1073/pnas.2105628118. 34385320 PMC8379959

[38] X. Wang , Z. Wang , F. P. García de Arquer , C.‐T. Dinh , A. Ozden , Y. C. Li , D.‐H. Nam , J. Li , Y.‐S. Liu , J. Wicks , Z. Chen , M. Chi , B. Chen , Y. Wang , J. Tam , J. Y. Howe , A. Proppe , P. Todorović , F. Li , T.‐T. Zhuang , C. M. Gabardo , A. R. Kirmani , C. McCallum , S.‐F. Hung , Y. Lum , M. Luo , Y. Min , A. Xu , C. P. O'Brien , et al., Nat. Energy 2020, 5, 478–486, 10.1038/s41560-020-0607-8.

[39] W. Sun , P. Wang , Y. Jiang , Z. Jiang , R. Long , Z. Chen , P. Song , T. Sheng , Z. Wu , Y. Xiong , Adv. Mater. 2022, 34, e2207691, 10.1002/adma.202207691. 36193772

[40] J. Zhou , B. He , P. Huang , D. Wang , Z. Zhuang , J. Xu , C. Pan , Y. Dong , D. Wang , Y. Wang , H. Huang , J. Zhang , Y. Zhu , Angew. Chem. Int. Ed. 2024, 64, e202418459. 10.1002/anie.202418459 39623792

[41] C. Chen , Z. Sun , G. Qin , B. Wang , M. Liu , Q. Liang , X. Li , R. Pang , Y. Guo , Y. Li , W. Chen , Adv. Mater. 2024, 36, e2409797, 10.1002/adma.202409797. 39370761

[42] X. P. Yang , Z. Z. Wu , Y. C. Li , S. P. Sun , Y. C. Zhang , J. W. Duanmu , P. G. Lu , X. L. Zhang , F. Y. Gao , Y. Yang , Y. H. Wang , P. C. Yu , S. K. Li , M. R. Gao , Nat. Commun. 2025, 16, 2811, 10.1038/s41467-025-58109-6. 40118841 PMC11928625

[43] N. Ye , K. Wang , Y. Tan , Z. Qian , H. Guo , C. Shang , Z. Lin , Q. Huang , Y. Liu , L. Li , Y. Gu , Y. Han , C. Zhou , M. Luo , S. Guo , Nat. Synth. 2025, 4, 799–807, 10.1038/s44160-025-00769-9.

[44] Y. Huang , Y. Gao , B. Zhang , Chem 2025, 11, 102533, 10.1016/j.chempr.2025.102533.

[45] J. Zhang , G. Zeng , S. Zhu , H. Tao , Y. Pan , W. Lai , J. Bao , C. Lian , D. Su , M. Shao , H. Huang , Proc. Natl. Acad. Sci. USA 2023, 120, e2218987120. 36877842 10.1073/pnas.2218987120 PMC10089218

[46] G. Zhang , Z. J. Zhao , D. Cheng , H. Li , J. Yu , Q. Wang , H. Gao , J. Guo , H. Wang , G. A. Ozin , T. Wang , J. Gong , Nat. Commun. 2021, 12, 5745, 10.1038/s41467-021-26053-w. 34593804 PMC8484611

[47] Y. Cao , Z. Chen , P. Li , A. Ozden , P. Ou , W. Ni , J. Abed , E. Shirzadi , J. Zhang , D. Sinton , J. Ge , E. H. Sargent , Nat. Commun. 2023, 14, 2387, 10.1038/s41467-023-37898-8. 37185342 PMC10130127

[48] T. Zhang , S. Xu , D. L. Chen , T. Luo , J. Zhou , L. Kong , J. Feng , J. Q. Lu , X. Weng , A. J. Wang , Z. Li , Y. Su , F. Yang , Angew. Chem. Int. Ed. 2024, 63, e202407748, 10.1002/anie.202407748. 38818639

[49] A. Xu , S.‐F. Hung , A. Cao , Z. Wang , N. Karmodak , J. E. Huang , Y. Yan , A. Sedighian Rasouli , A. Ozden , F.‐Y. Wu , Z.‐Y. Lin , H.‐J. Tsai , T.‐J. Lee , F. Li , M. Luo , Y. Wang , X. Wang , J. Abed , Z. Wang , D.‐H. Nam , Y. C. Li , A. H. Ip , D. Sinton , C. Dong , E. H. Sargent , Nat. Catal. 2022, 5, 1081–1088, 10.1038/s41929-022-00880-6.

[50] W. Guo , X. Yao , L. Peng , B. Lin , Y. Kang , L. Gan , Chin. Chem. Lett. 2020, 31, 836–840, 10.1016/j.cclet.2019.06.018.

[51] K. Fan , W. Xie , J. Li , Y. Sun , P. Xu , Y. Tang , Z. Li , M. Shao , Nat. Commun. 2022, 13, 7958, 10.1038/s41467-022-35664-w. 36575160 PMC9794814

[52] Y. Liu , J. Wei , Z. Yang , L. Zheng , J. Zhao , Z. Song , Y. Zhou , J. Cheng , J. Meng , Z. Geng , J. Zeng , Nat. Commun. 2024, 15, 3619, 10.1038/s41467-024-48035-4. 38684692 PMC11059385

[53] K. Liu , H. Li , M. Xie , P. Wang , Z. Jin , Y. Liu , M. Zhou , P. Li , G. Yu , J. Am. Chem. Soc. 2024, 146, 7779–7790, 10.1021/jacs.4c00429. 38466142

[54] Y. Li , Y. Tan , M. Zhang , J. Hu , Z. Chen , L. Su , J. Li , Angew. Chem. Int. Ed. 2024, 63, e202411068. 10.1002/anie.202411068 39137126

