# Supplementary material for: Local Coordination‐Dependent CO2 Reduction Activity of Bimetallic Cu─Al Catalysts for Selective Ethylene/Ethanol Electrosynthesis
Source: Angew Chem Int Ed Engl. 2025 Nov 10;65(2):e20291. doi: 10.1002/anie.202520291 (PMC12790349; doi:10.1002/anie.202520291)
Supplement: Supplementary file 1 — Supporting Information [file ANIE-65-e20291-s001.pdf]

Supporting Information

©Wiley-VCH 2021

69451 Weinheim, Germany

Local coordination-dependent CO_2 reduction activity of bimetallic Cu–Al catalysts for selective ethylene/ethanol electrosynthesis

Weihua Guo,‡[a][b] Xingyu Wang,‡[c] Yangbo Ma,‡[d] Yun Song,[a][b] Liang Chang,[e] Haoran Wu,[f] Geng Li,[a][b] Zhihao Li,[a][b] Yinger Xin,[a][b] MingMing He,[a][b] Jixun Zhang,[g] Tao Yang,[g] Minghui Zhu,[f] Hanchen Shen,[h] Shibo Xi,[i] Xue Wang,[j] Lin Gan[e], Qiu Jiang,[k] Chuang Xia,[k] Shenlong Zhao,[l] Zhengxiao Guo,*[d] Ziyun Wang,*[c] Ben Zhong Tang,[h] Ruquan Ye,*[a][b]

Abstract: Cu-based catalysts are optimal for the electroreduction of CO_2 to hydrocarbon products. The formation of bimetallic catalysts has been widely adopted to improve product selectivity. However, discrepancies in product distribution in the literature, even among catalysts with identical bimetal compositions, suggest the involvement of distinct reaction pathways. Here we report that ethylene and ethanol selectivity are strongly influenced by the atomic coordination of metals. We prepared two model catalysts to investigate this phenomenon, namely interface-CuAl (dominated by Cu/$CuAlO_2$ interfaces) and doping-CuAl (with Al doped into the Cu lattice). Both catalysts demonstrate excellent C_{2+} Faradaic efficiency (FE) of 65-85%, consistent with the alloy effect. However, the structural differences result in pronounced variations in product selectivity. Over the interface-CuAl, ethylene is the primary product with an FE of 67.6%, which is seven-fold higher than $FE_{ethanol}$. Conversely, doping-CuAl favors ethanol production, reaching a maximum $FE_{ethanol}$ of 43.7%, four times higher than ethylene. Extended X-ray absorption fine structure and *in situ* Fourier transform infrared spectrometry reveal that distinct adsorption abilities of Cu active sites influence the reaction pathway, causing different intermediate coverages. Complementary theoretical calculation further elucidates the reaction mechanism, highlighting the critical role of the bifurcation of *CHCOH intermediate. Specifically, favorable C-O cleavage at interface-CuAl promotes ethylene production, whereas Cu-C scission at doping-CuAl favors ethanol production. Beyond CO_2 electroreduction, the CuAl catalysts were also evaluated for nitrate reduction, where their phase-dependent activity further underscores the importance of atomic coordination in governing catalytic performance. This study provides fundamental insights into the structure-selectivity relationship of Cu-based bimetallic catalysts for selective chemical production.

This PDF file includes:

Supplementary Text
Figures. S1 to S42
Tables S1 to S4
References

Experimental Section

Material synthesis

Preparation of interface-CuAl and doping-CuAl. A pulsed Nd:YAG laser (Nimma-600, Beamtech) was employed to ablate a CuAl target immersed in deionized water for the synthesis of interface-CuAl. The laser parameters were set as follows: pulse width 7 fs, wavelength 1,064 nm, pulse frequency 15 Hz. The raw CuAl ratio with 1:1 and 3:1 material of 99.99% purity was made into a square target 20 mm in length and 5 mm in thickness. Before the laser ablation, the target was placed in a 50 ml beaker containing deionized water, with water surface was 10 mm higher than the target surface. The laser ablation lasted for 60 min to obtain a colloid solution with CuAl NPs, which was collected by a centrifuge at 10,000 rpm for 15 min three times and then dried in a vacuum oven to obtain the CuAl powder.

Preparation of $CuAlO_2$. $CuAlO_2$ was synthesized via a sol-gel method. Specifically, 7 mmol of $Al(NO_3)_3 \cdot 9H2O$ and 7 mmol of $Cu(NO_3)_2 \cdot 3H_2O$ were dissolved in 30 mL of methanol under stirring to form a clear solution. Then, 28 mmol of $C_6H_8O_7 \cdot H_2O$ dissolved in 30 mL of deionized water was added. The resulting mixture was stirred under the same conditions until uniform, followed by gelation under vigorous stirring at 70 °C for 24 h. The resulting gel was collected and further dried in an oven at 120 °C for 2 hours. The dried gel was then calcined in a muffle furnace at 1150 °C for 4 hours. Finally, the product was cooled to room temperature, yielding $CuAlO_2$.

Characterizations The high-angle annular dark-field scanning transmission electron microscopy (HAADF-STEM) combined with energy-dispersive X-ray spectroscopy (EDX) was employed to record the STEM and mapping images at an accelerating voltage of 200kV. The X-ray diffraction (XRD) patterns of the samples were measured by a Bruker D2 using a Cu Kα source, with a scan step of 10° $min^{-1}$and a scan range between 10°−80°. The X-ray photoelectron spectroscopy (XPS) data were collected on a Thermo ESCALAB 250Xi spectrometer equipped with a monochromatic AlK radiation source (1486.6 eV, pass energy 20.0 eV). The data were calibrated with C 1s 284.8 eV. X-ray absorption fine structure (XAFS) measurements were performed in the transmission mode at beamline X-ray absorption fine structure for catalysis of the Singapore Synchrotron Light Source operated at 700 MeV with a beam current of 200 mA. Data processing was performed using the Athena and Artemis software packages. The in situ electrochemical FTIR measurements were performed by using a Thermo iS50. The online DEMS was provided by online analysis of produced intermediates and products of catalysts during CO_2RR in a flow cell.

Electrochemical measurements

Electrochemical reduction of carbon dioxide in a flow cell. CO_2 reduction was conducted in a three-chamber flow cell. The CO_2 gas was supplied directly to the catalyst layer (cathode, working electrode). The CO_2 gas flow rate was controlled using a mass flow controller and set to 10 sccm and 20 sccm for interface-CuAl and doping-CuAl, respectively. 1 M KOH solution was used as both the catholyte and the anolyte. A platinum plate was used as the anode (counter electrode). Peristaltic pumps were used to control the flow rate of the electrolytes at ~10 ml min^{-1}. An AEM (FAA-3-PK-75, Fumatech) was used to separate the cathode and anode chambers. Electrolysis experiments were conducted using chronoamperometry by a CHI 650 electrochemical workstation (Chenhua, Shanghai). The cathode potentials were measured against an Ag/AgCl reference electrode (1 M KOH, Gaossunion Co., Ltd., Tianjin). It should be noted that iR correction was not performed. All the potentials are without iR correction to avoid large systematic errors under high currents.

Electrochemical reduction of potassium nitrate in an H-cell. All NITRR experiments were carried out using a three-electrode system in a two-compartment H-cell separated by an ion-exchange membrane (Nafion 117) that connected to a CHI 650 electrochemical workstation (Chenhua, Shanghai). The obtained catalysts loading on 0.5 cm^2 glass carbon, Hg/HgO, and platinum plate were used as the working electrode, reference electrode, and counter electrode, respectively. 15mL mixed KOH/KNO_3 solution (with different configurations) was used as the cathode electrolyte and anode electrolyte. All potentials were recorded against the reversible hydrogen electrode (RHE) using $E_{RHE} = E_{Hg/HgO} + 0.0591 * pH + 0.098$. Cyclic voltammetry (CV) and LSV were performed at a scan rate of 10 mVs^{-1} and 5 mV s^{-1}, respectively. The potentiostatic tests were carried out at different potentials for 3600 s with a stirring rate of 800 rpm. The potential range for measuring the NH_3 FEs and yield rates was from −0.5 V to −1.0 V vs. RHE with intervals of −0.1 V. The isotopic labeling experiments were carried out using the same methods at −0.8 V vs. RHE, except the N-source was replaced by 99% $^{15}NO_3^-$. Unless otherwise specified, all measurements were carried out in ambient conditions without iR-compensation.

Product detection.

The gas and liquid products under different potentials during CO_2RR and NITRR were characterized through the online gas chromatograph (GC) and nuclear magnetic resonance (NMR), respectively.

Ammonia detection. ^{1}H nuclear magnetic resonance (^{1}H NMR) was recorded on an AVANCE III HD 300 system to detect ammonia. The pH value of the final electrolyte was adjusted to be weakly acidic with 2 M HCl. Maleic acid ($C_4H_4O_4$, 50 ppm) was employed as the external standard to calibrate the standard curve of NH_4^+ using the peak area ratio between NH_4^+ and maleic acid. The isotope labeling experiments were also measured using the same process.

Nitrite detection. The nitrite concentration was measured by UV–vis spectrophotometry according to the standard method. First, a mixture of p-aminobenzene sulfonamide (4 g), N-(1-naphthyl) ethylenediamine dihydrochloride (0.2 g), deionized water (50 ml) and phosphoric acid (10 mL, ρ = 1.685 g ml^{-1}) was used as the color reagent. The electrolyte sample was collected

and diluted to the detection range. Next, 40 μl of the color reagent was added into the 2.0 ml sample solution, mixed thoroughly, and rested for 20 min at ambient conditions. The absorption intensity at a wavelength of 540 nm was then tested by UV–vis spectrophotometry (UV-2600). The concentration–absorbance curve was calibrated using a series of standard potassium nitrite solutions with linear fitting prepared beforehand. Nitrite product concentrations were then calculated based on the tested absorbance and the standard curveThe Faradaic efficiency (FE) and yield were calculated according to the following equations:

$$FE_{gas} = \frac{Q_{gas}}{Q_{total}} \times 100\% = \frac{n_{gas} \times N \times F}{j \times t} \times 100\% = \frac{\frac{P}{RT} \times N \times F}{j} \times v \times 100\%$$

$$FE_{liquid} = \frac{Q_{liquid}}{Q_{total}} \times 100\% = \frac{n_{liquid} \times N \times F}{j \times t} \times 100\%$$

$$Yield_{NH3\ or\ C2H4} = \frac{n_{NH3\ or\ C2H4} \times M_{NH3\ or\ C2H4}}{m_{Cu} \times t}$$

n is the amount of product (mol), N is the number of electrons transferred to form a molecule of product; F = 95200 C mol^{-1} is Faraday constant, P is the atmosphere pressure (Pa), T = 298 K is the temperature (K) and R is the molar gas constant =8.31 J $(mol\ K)^{-1}$, v is Gas flow rate, j is the total current, t is the electrolysis time (s), M is the Molar mass of product (g mol^{-1}), m is the quality of copper (mg).

OH^- electro adsorption measurements. In-situ OH_{ads} studies were conducted by flowing Ar in the H-cell. First, CO_2 electrolysis was conducted at a constant potential of −0.5 V versus the RHE for 30 min by switching the gas feed to CO_2. Immediately after electrolysis, the gas feed was switched to Ar, and then cyclic voltammetry (20 mV s^{-1}) was performed.

In-situ FTIR spectroscopy. In-situ FTIR spectra were acquired in a three-electrode cell equipped with a Thermo Scientific Nicolet iS50. Ag/AgCl and Pt wire were used as the reference electrode and counter electrode, respectively. 0.1 M CO_2-saturated $KHCO_3$ was taken as the electrolyte. Each spectrum was recorded by 32 scans at an 8 cm^{-1} spectral resolution.

Online DEMS. A flow cell was used during the DEMS measurement. The i-t technique was applied for 10 minutes after the baseline was stabilized. The corresponding mass signal then appeared. After the electrochemical test was finished and the mass signal returned to the baseline, the next cycle was started using the same test conditions to avoid unexpected errors during the DEMS measurement. After four cycles, the experiment was finished.

Computational details

Density functional theory (DFT) calculations were executed utilizing the Vienna Ab-initio Simulation Package (VASP)[1]. The generalized gradient approximation (GGA) within the Perdew-Burke-Ernzerhof (PBE) functional framework was employed to describe the electron exchange and correlation energy[2]. The interaction between core and valence electrons was treated using the frozen-core projector augmented-wave (PAW) method with an energy cutoff of 450 eV[3]. For geometry optimization, the convergence criteria for energy and force were set at 1.0 × 10^{-5} eV per atom and 0.05 eV $Å^{-1}$, respectively. Structural optimizations were performed using a Γ-centered Monkhorst-Pack k-point mesh of 3×3×1[4]. The free energy of the intermediates is defined as follows:

$$\Delta G = \Delta E + \Delta E_{ZPE} - T\,\Delta S$$

In this context, ΔE denotes the reaction energy for each step, as determined by DFT calculations. ΔE_{ZPE} represents the variation in zero-point energies throughout the reactions. The term TΔS accounts for the entropy contribution at a temperature of 298.15 K, where ΔS signifies the entropy difference between the adsorbed state and the gas phase. Additionally, the energy of a proton/electron pair is defined as half the energy of a hydrogen molecule, following the principles of the computational hydrogen electrode (CHE) method[5].

Adsorption Energy

The adsorption energy (ΔE_{ads}) of the key CO_2RR intermediates, including *CHCOH and *CCH, was calculated relative to CO_2, H_2 and H_2O under conditions of T = 298.15 K according to following equations:

$$\Delta E_{*CCH} = E_{*CCH} - 4.5{*}\,E_{H2} – E{*} –2{*}\,E_{CO2} + 4{*}E_{H2O}$$

$$\Delta E_{*H} = E_{*H} - E{*} -2{*}\,E_{CO2} + 3{*}E_{H2O} - 4.5{*}\,E_{H2}$$

Where * represents the adsorption sites.

Models

The CuAl slab was constructed based on a Cu(111) surface. Starting from a Cu bulk structure, Al atoms were substituted for Cu atoms in a 3:1 ratio (Cu:Al). The resulting structure was then expanded to a 2×2×1 supercell. The final slab model had lattice dimensions of 10.28×10.28×21.29 Å^3. To prevent artificial interactions between periodic images in the z-direction, a vacuum space of 15 Å was added above the surface. The model contained a total of 64 atoms in the slab. The $CuAlO_2$/Cu model was constructed by depositing a Cu cluster on the $CuAlO_2$(100) surface. The substrate was based on the $CuAlO_2$(100) surface, while the Cu cluster consisted of nine atoms arranged in a configuration mimicking the Cu(111) surface structure. The Cu9 cluster was placed on top of the $CuAlO_2$(100) surface to simulate the interface between $CuAlO_2$ and Cu. A vacuum space of 15 Å was added in the z-direction to prevent artificial interactions between periodic images. The total dimensions of the simulation cell were 11.37×11.30×24.03 Å^3. The model contained a total of 137 atoms in the slab.

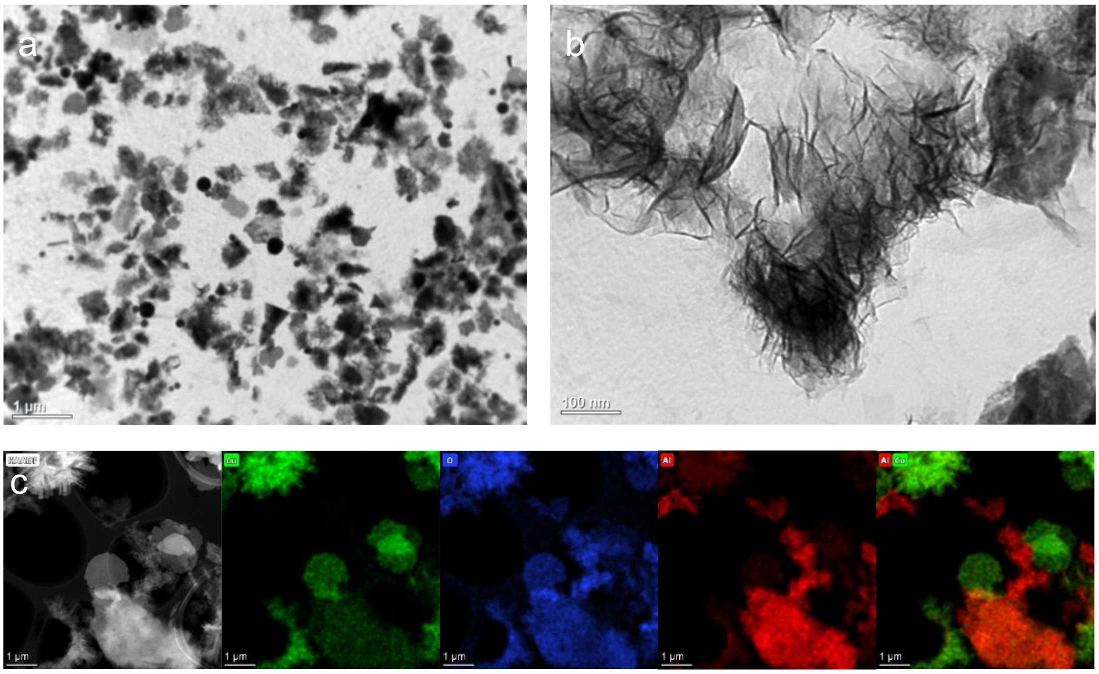

Figure S1. (a, b) TEM images of interface-CuAl. (c) Typical EDS spectra of interface-CuAl.

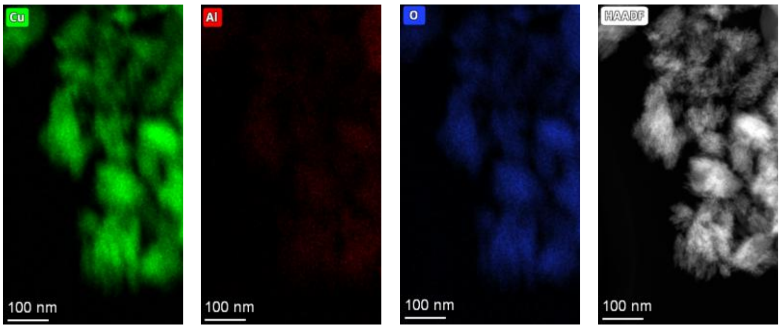

Figure S2. Typical EDS spectra of doping-CuAl.

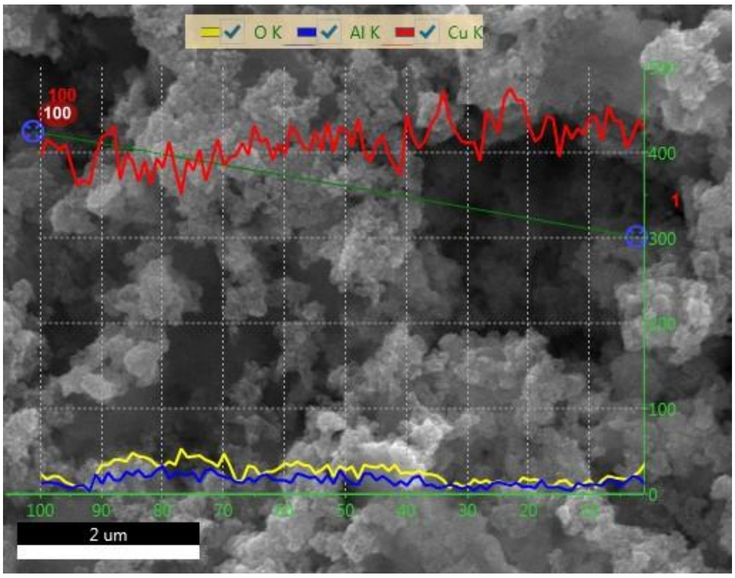

Figure S3. Structural characterization of doping-CuAl. EDX line scan of reductive doping-CuAl during CRR process.

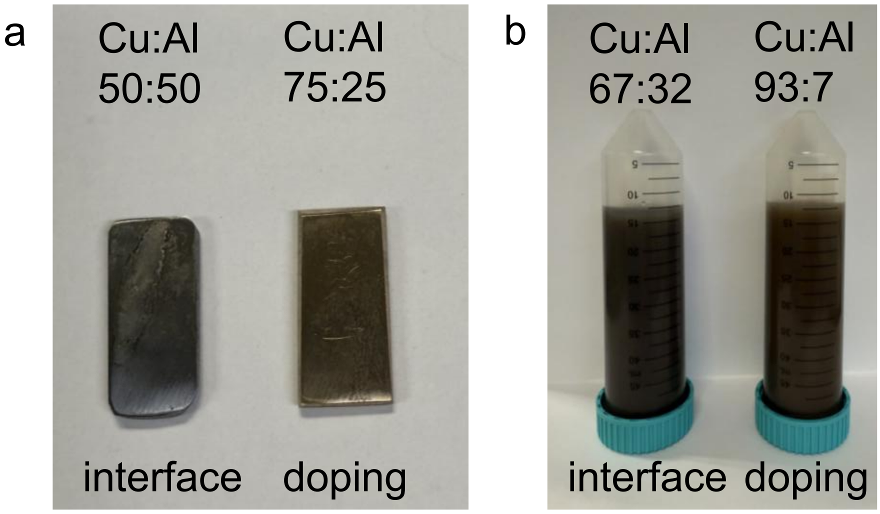

Figure S4. (a) Target materials with different copper-aluminum ratios (Cu:Al, 1:1 and 3:1). (b) After the laser was applied, catalyst solutions with different copper-aluminum ratios were collected and washed three times with ethanol to removal excess aluminum oxide.

After laser, the decrease of Al element may be due to the Cu absorbs laser energy in this band more easily than aluminum during the laser process, so more copper atoms enter the solution to form a catalyst compared with Al.

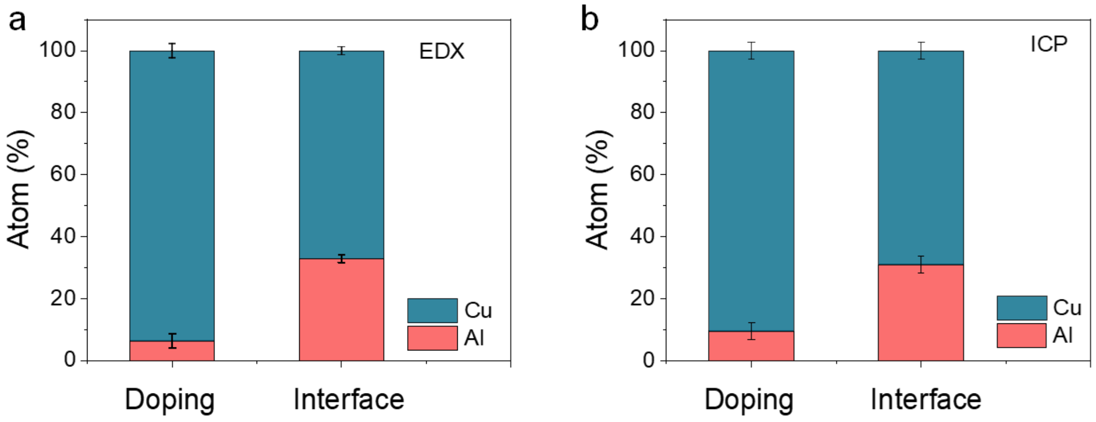

Figure S5. Typical atom ratio Cu/Al of doping-CuAl and interface-CuAl.

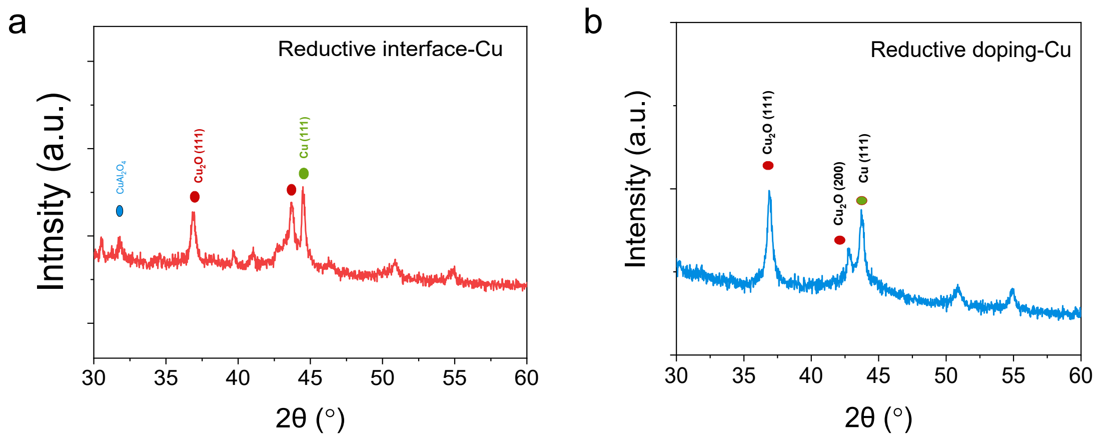

Figure S6. Structural characterization of Interface and Doping. XRD pattern of (a) reductive interface-CuAl and (b) reductive doping-CuAl during CRR process.

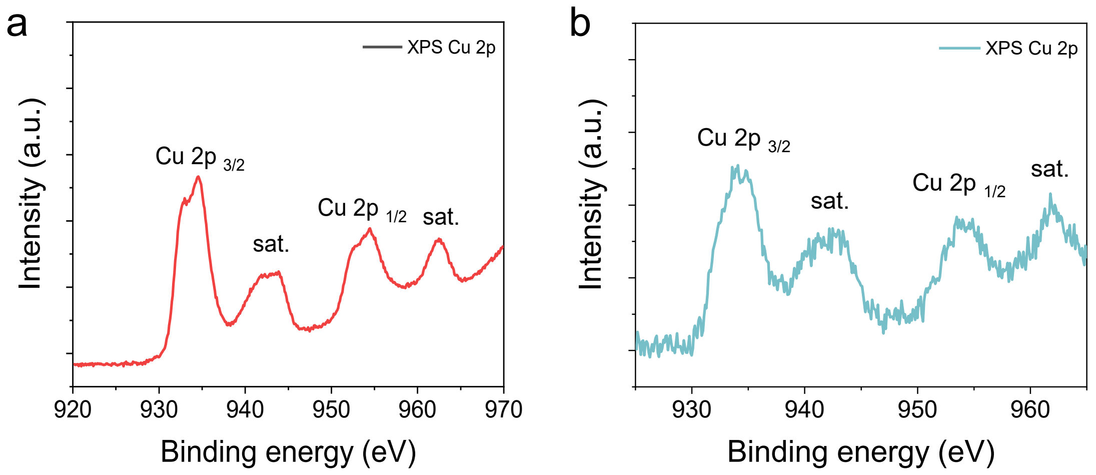

Figure S7. Structural characterization of interface-CuAl and doping-CuAl. Full spectrum Cu 2p XPS spectra of (a) interface-CuAl and (b) doping-CuAl.

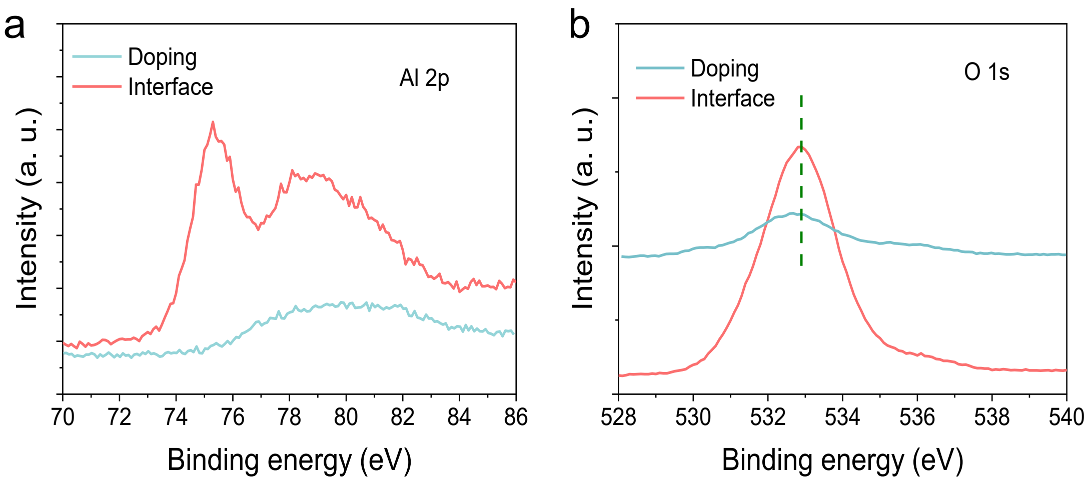

Figure S8. Structural characterization of interface-CuAl and doping-CuAl. XPS spectrum (a) Al 2p XPS spectra and O 1s spectra in interface-CuAl and doping-CuAl.

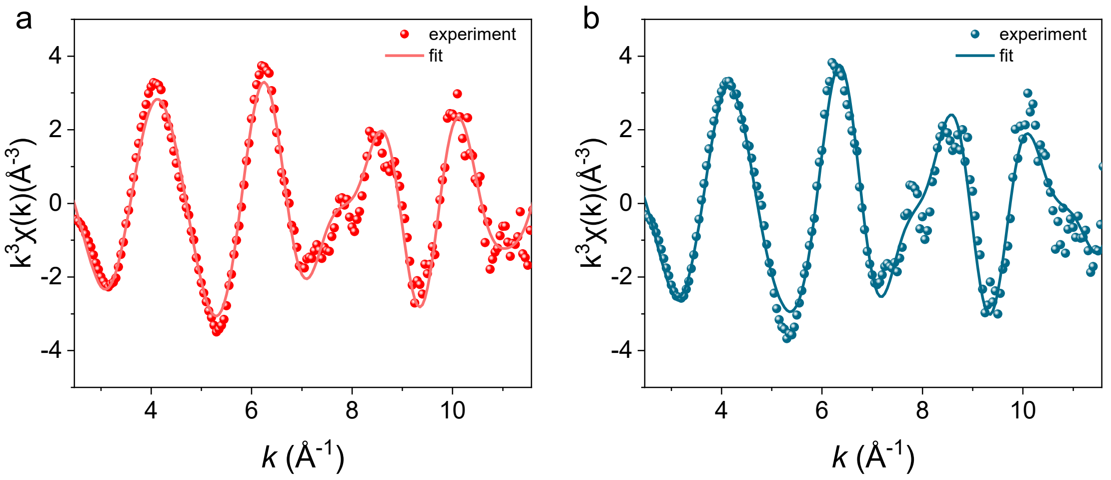

Figure S9. Fitting results of Cu K-edge spectra of different catalysts. Fourier transform of Cu K-edge EXAFS fitting results of (a) interface-CuAl and (b) doping-CuAl, respectively.

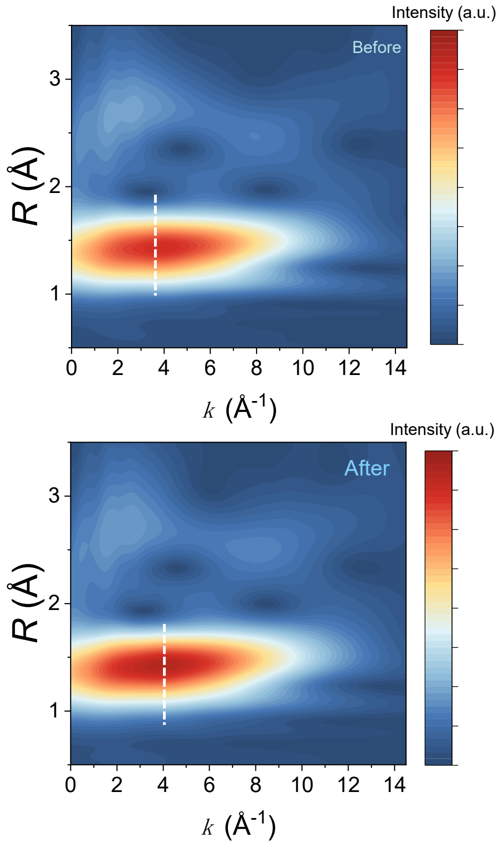

Figure S10. Characterizations. WT–EXAFS Cu K-edge spectra of the interface-CuAl catalyst before and after CO_2RR test.

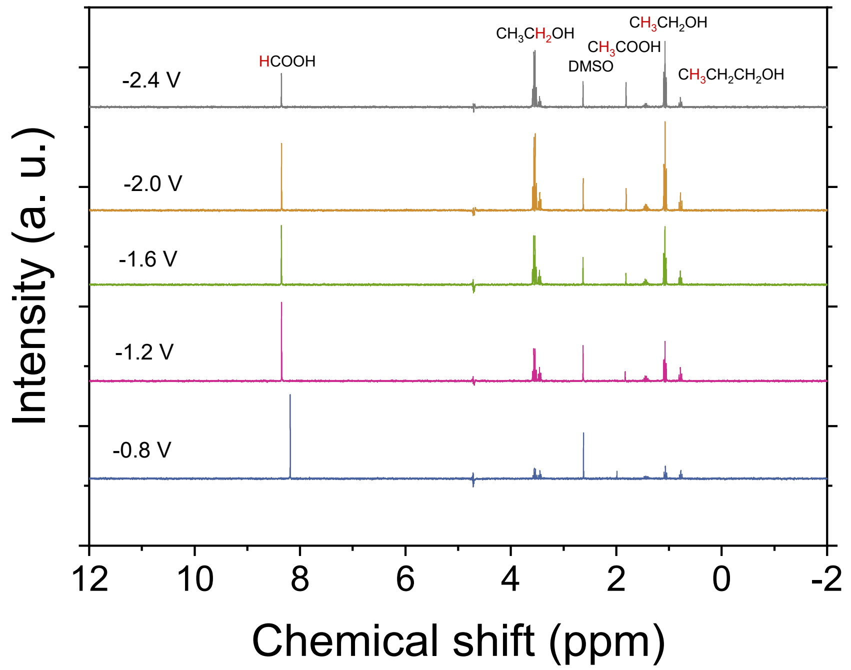

Figure S11. Product analysis. Representative 1H-NMR spectrum of liquid products collected from the cathode side in doping-CuAl.

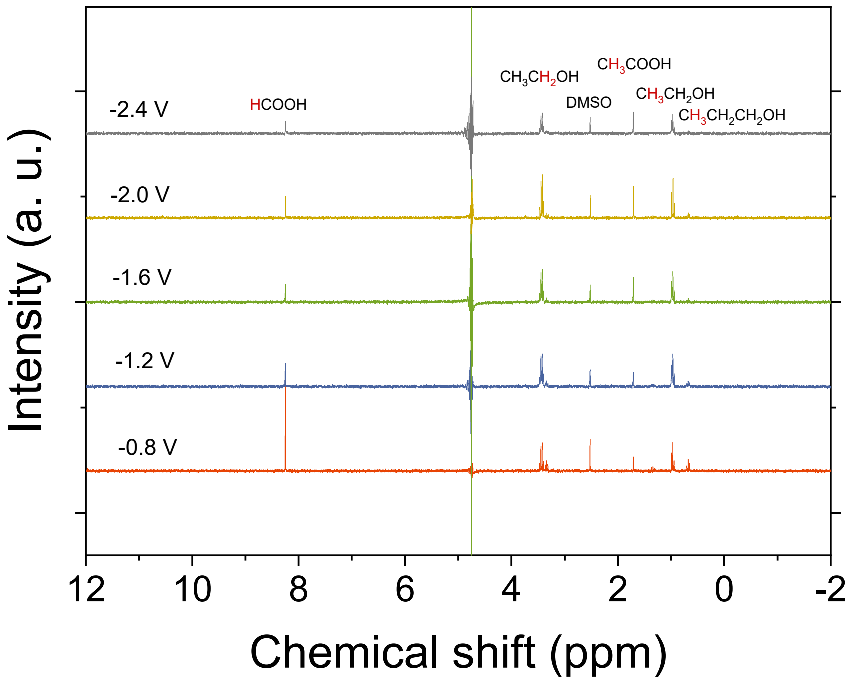

Figure S12. Product analysis. Representative 1H-NMR spectrum of liquid products collected from the cathode side in interface-CuAl.

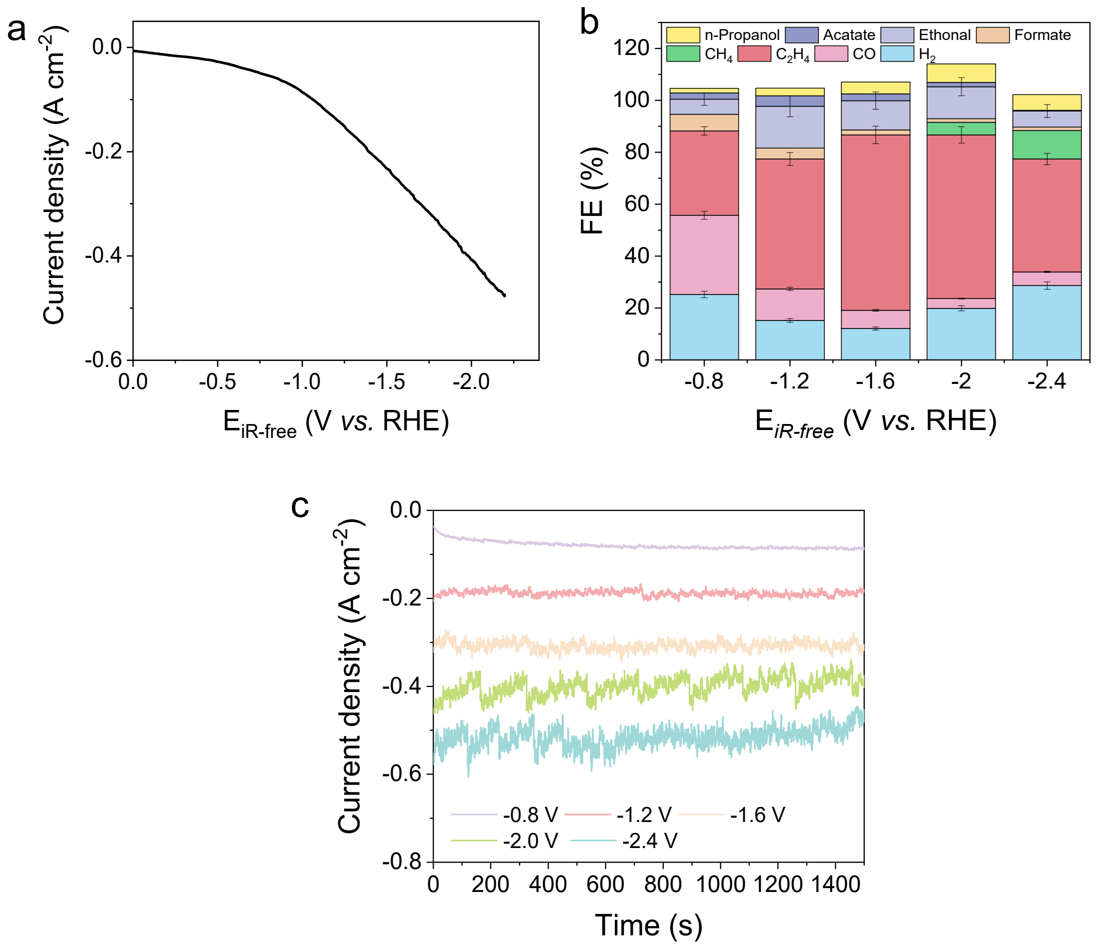

Figure S13. Electrochemical performances. (a) Linear scan voltammetry curve, (b) FE products and (c) chronoamperometry of interface-CuAl at different potential during CO_2RR.

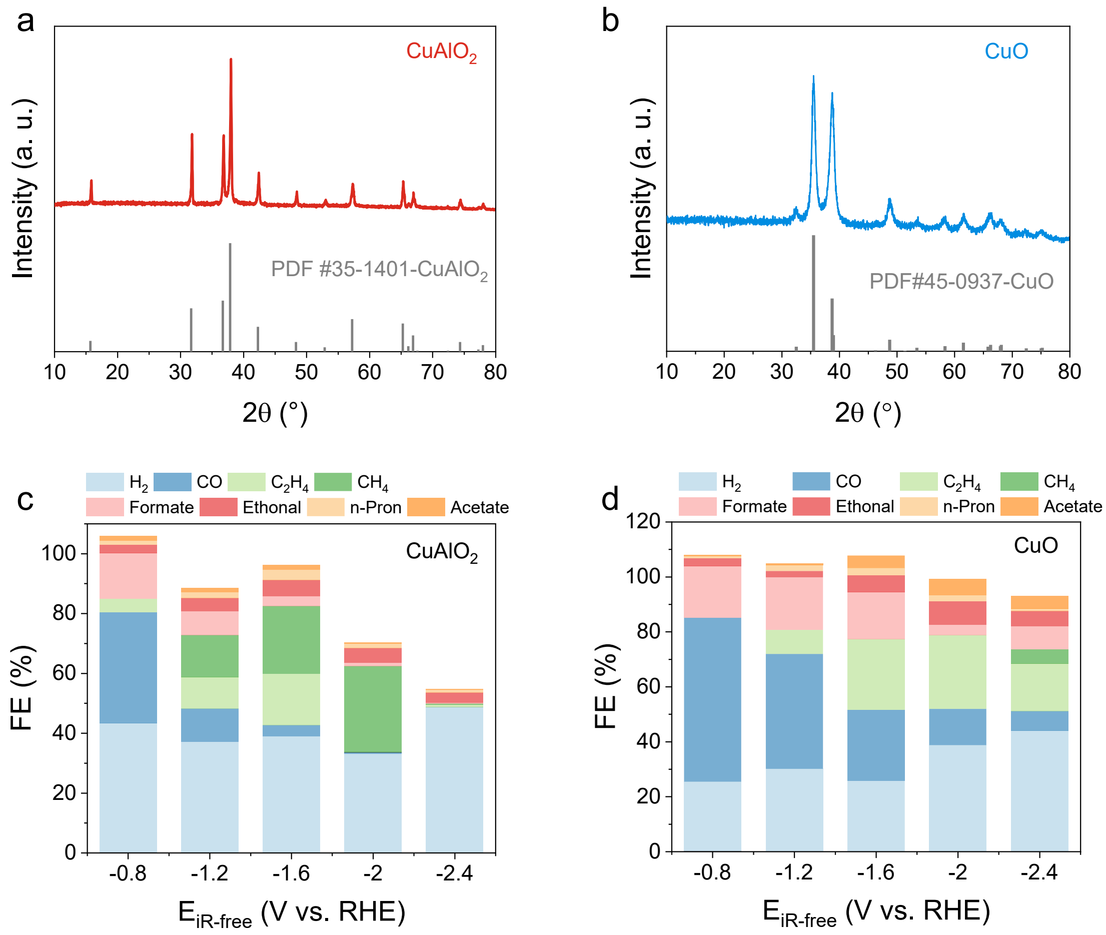

Figure S14. Electrochemical performances. The XRD pattern of (a) $CuAlO_2$ and (b) Commercial-CuO. FE products (c) $CuAlO_2$ and (d) Commercial-CuO at different potential during CO_2RR.

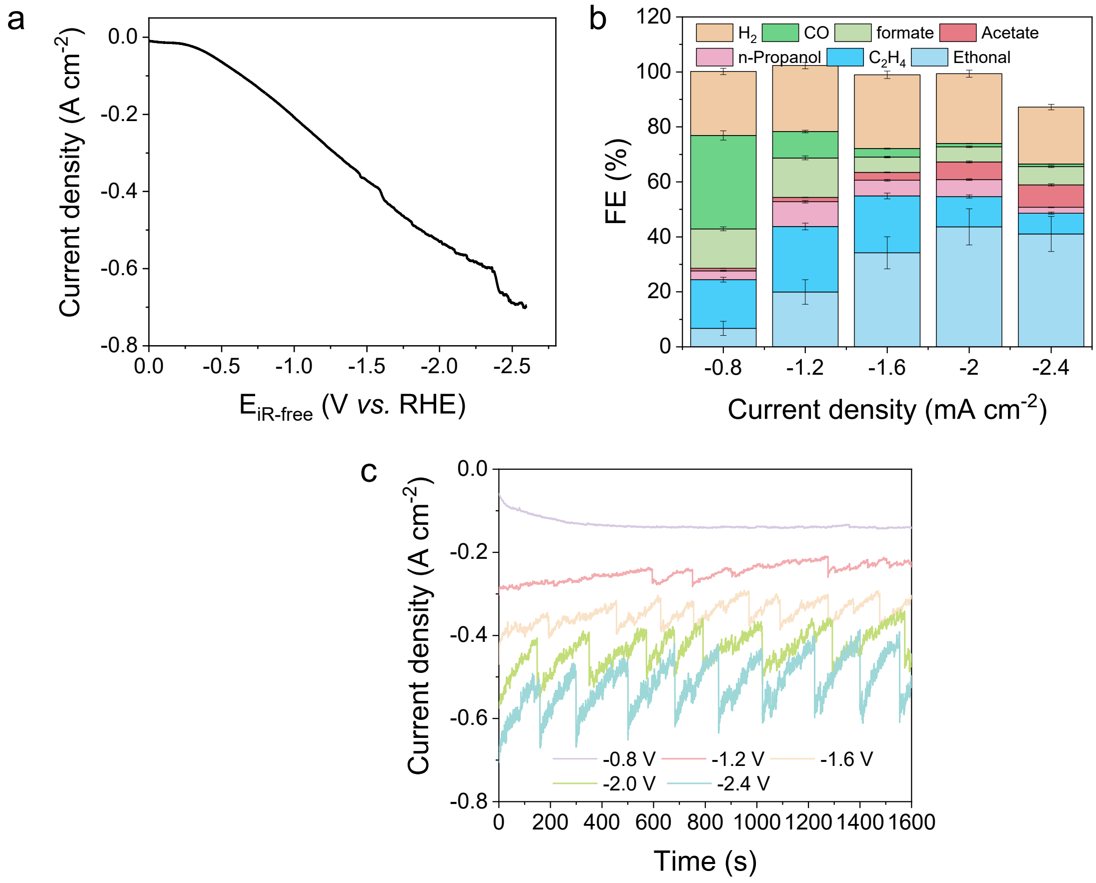

Figure S15. Electrochemical performances. (a) Linear scan voltammetry curve, (b) FE products and (c) chronoamperometry of doping-CuAl at different potential during CO_2RR.

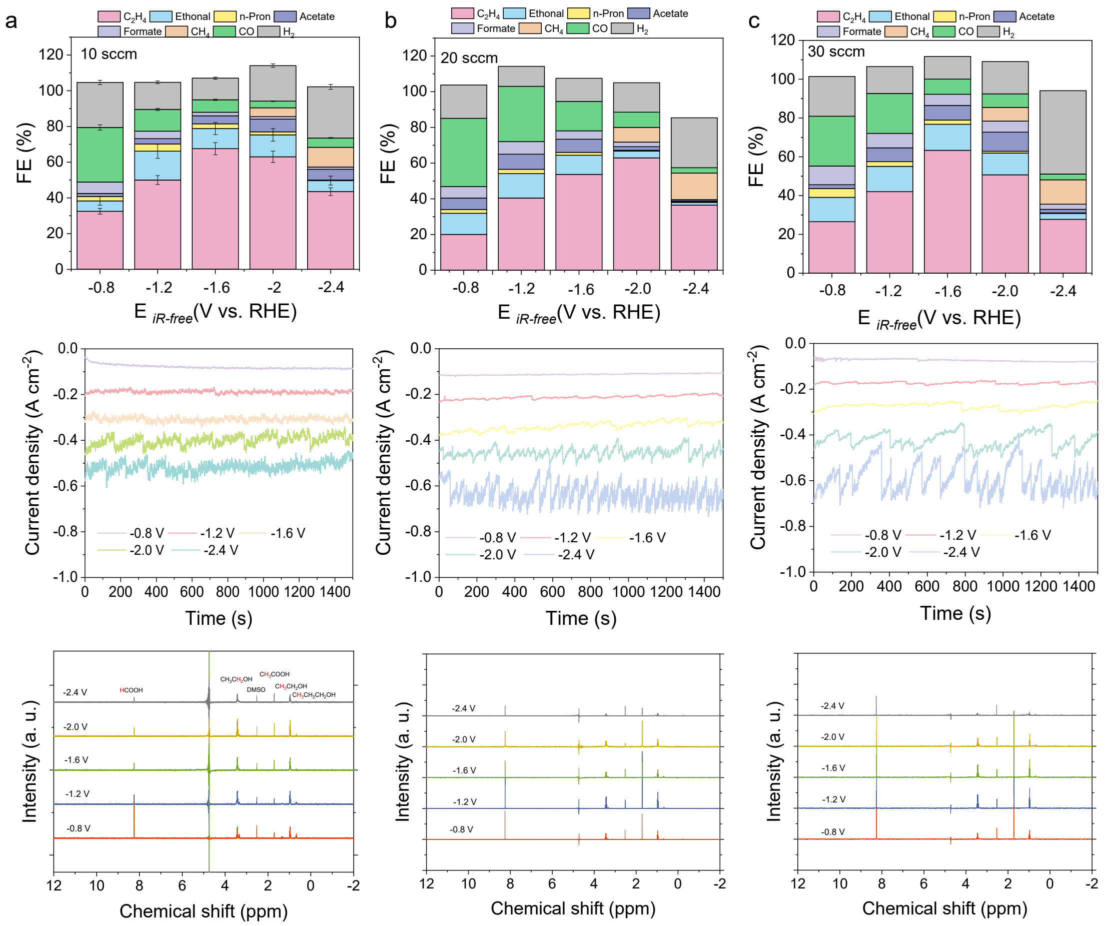

Figure S16. Electrochemical performances. FE, it and NMR results of different products during CO_2RR of interface-CuAl in different CO_2 flow rate (a) 10 sccm, (b) 20 sccm and (c) 30 sccm.

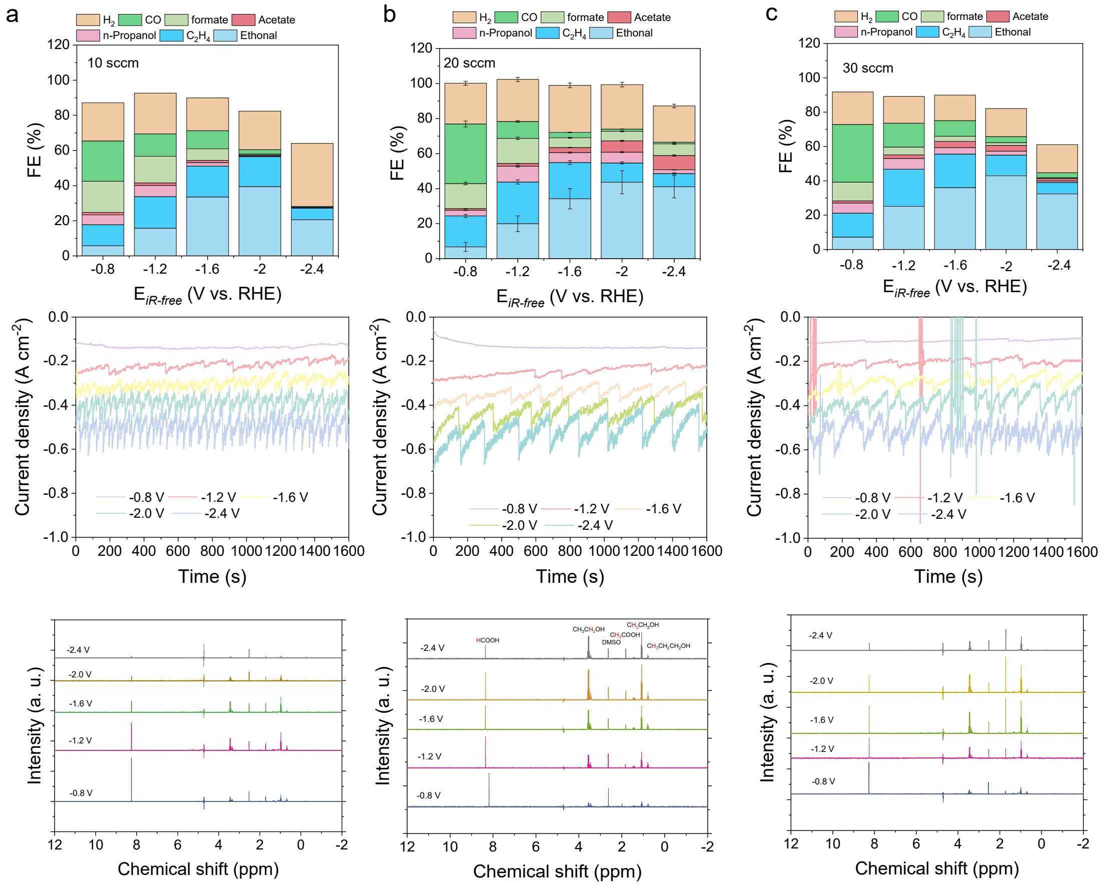

Figure S17. Electrochemical performances. FE, it and NMR results of different products during CO_2RR of doping-CuAl in different CO_2 flow rate (a) 10 sccm, (b) 20 sccm and (c) 30 sccm.

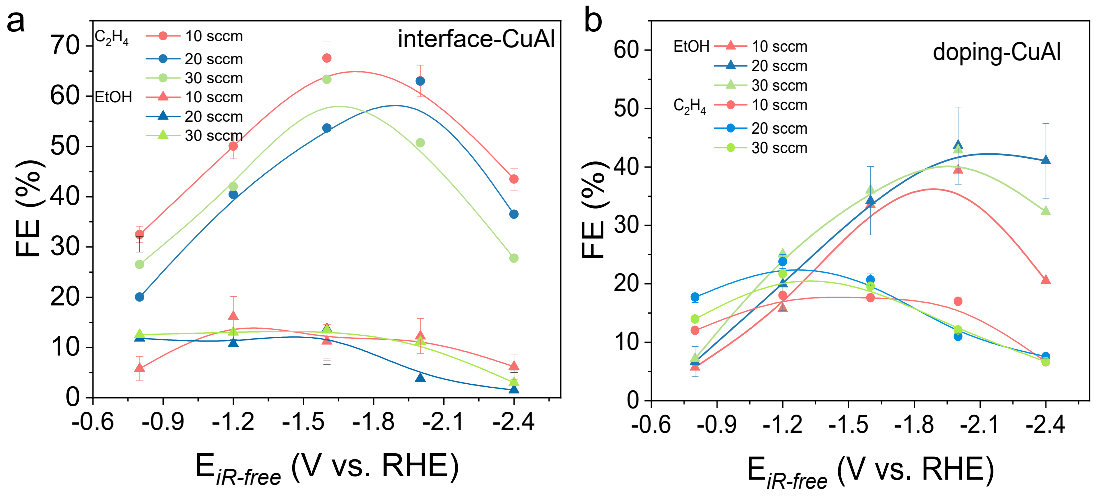

Figure S18. Electrochemical performances. FE comparison of C_2H_4 and ethanol during CO_2RR in different CO_2 flow rates of interface-CuAl (a) and doping-CuAl (b).

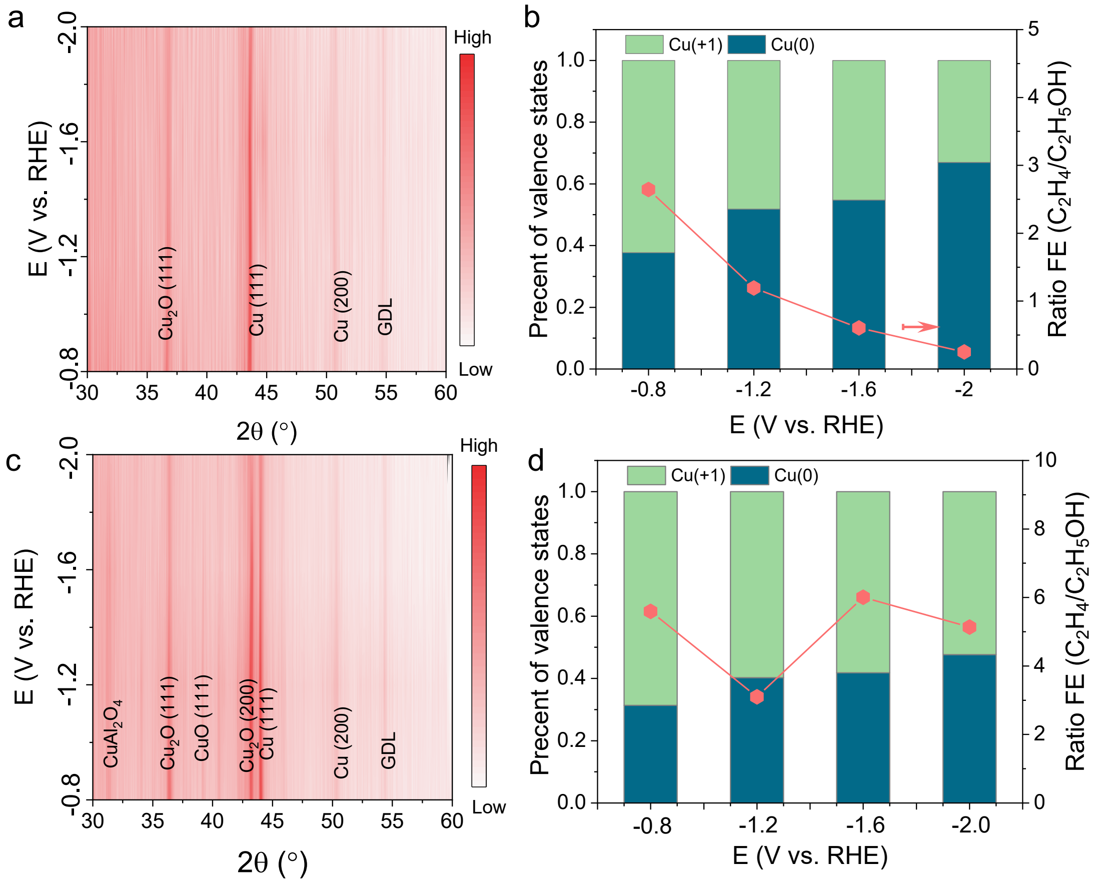

Figure S19. Structural evolution. Ex-situ XRD result of (a) doping-CuAl and (b) interface-CuAl during CO_2RR process. The relationship between the valence states of Cu and the ratio of FE of C_2H_4/C_2H_5OH on (b) doping-CuAl and (d) interface-CuAl.

In the CO_2RR process, the valence state of Cu in doped CuAl is mainly 0 and +1. As the potential increases, the valence state of Cu changes slightly, and more Cu(0) is generated. Combined with the electrochemical measurement results, it is further found that as the Cu(0)/Cu(+1) ratio increases, the C_2H_4/C_2H_5OH ratio decreases, indicating that the ratio of Cu oxidation state affects the product selectivity. Meanwhile, this conclusion is also proved in interface-CuAl. With the increase of potential, the ratio of Cu(0) to Cu(+1) in interface-CuAl increases slightly, but Cu(+1) still dominates. Combining with electrochemical measurements, the C_2H_4 is the main product

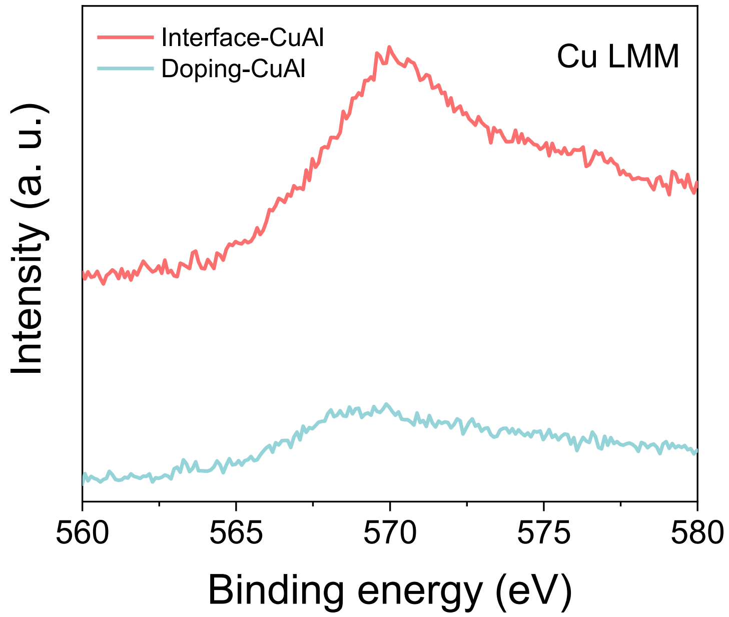

Figure S20. Characterizations of the tested interface-CuAl and doping-CuAl. Cu LMM XPS spectra of doping-CuAl and interface-CuAl after CO_2RR test.

The oxidation of Cu can also be inferred from the Cu LMM (Figure S20), which shows that the Cu LMM peak of interface-CuAl is at higher binding energy, implying an average higher oxidation than doping-CuAl.

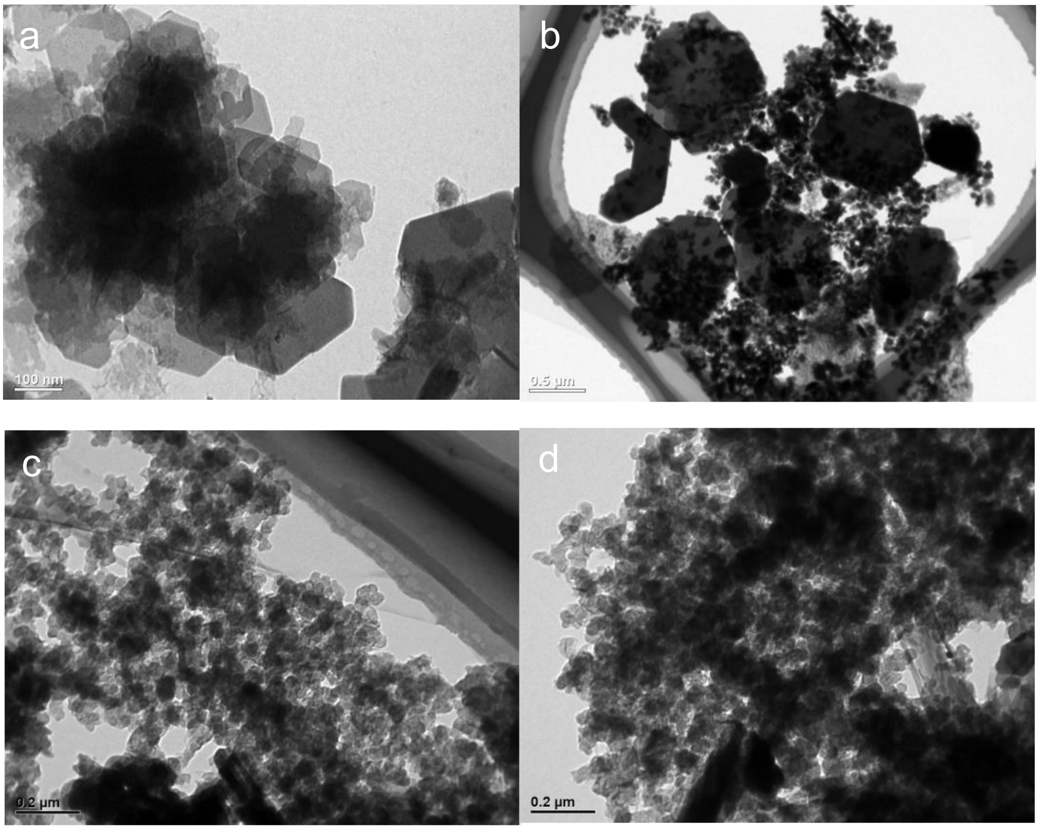

Figure S21. Characterizations of the tested interface-CuAl and doping-CuAl. TEM of (a) interface-CuAl and (b) doping-CuAl after CO_2RR test.

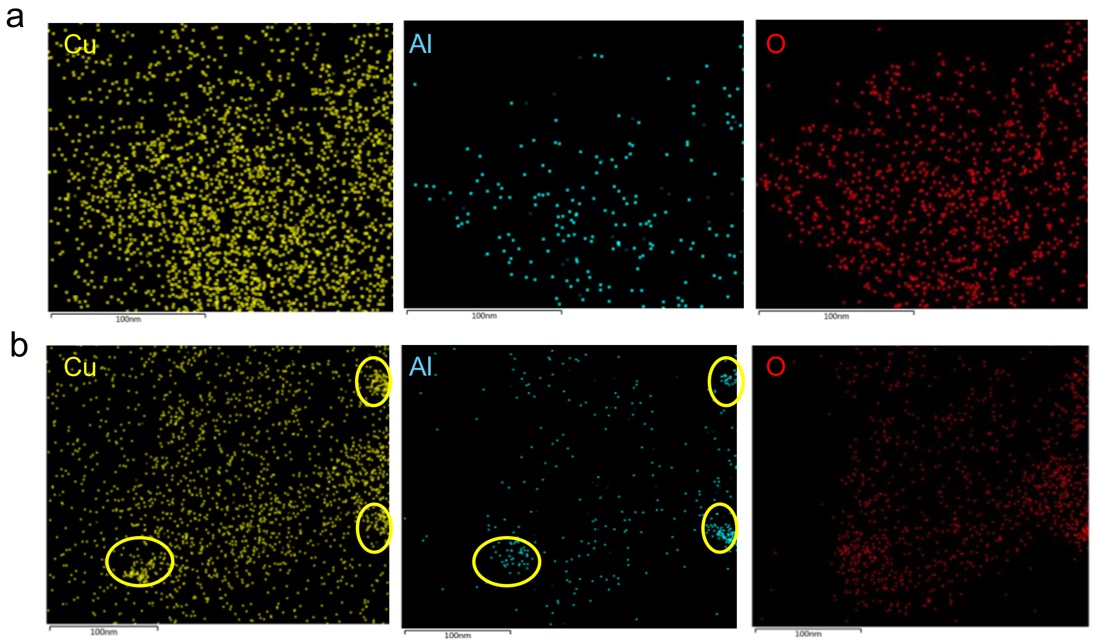

Figure S22. Characterizations of the tested interface-CuAl and doping-CuAl. EDX-mapping of (a) doping-CuAl and (b) interface-CuAl after CO_2RR test. Yellow Cu, blue Al and red O. yellow circle: $CuAl_2O_4$.

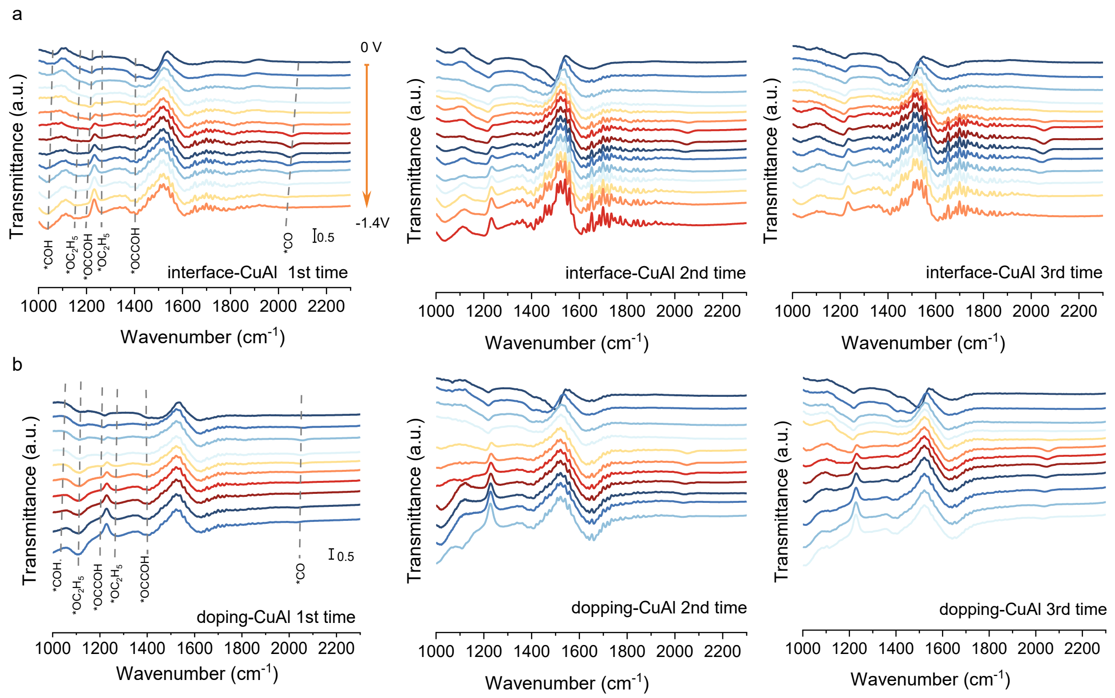

Figure S23. In-situ ATR-FTIRS measurement. In situ ATR-FTIRS obtained during chronopotentiometry repeat 3 times for interface-CuAl (a) and doping-CuAl (b) under CO_2RR.

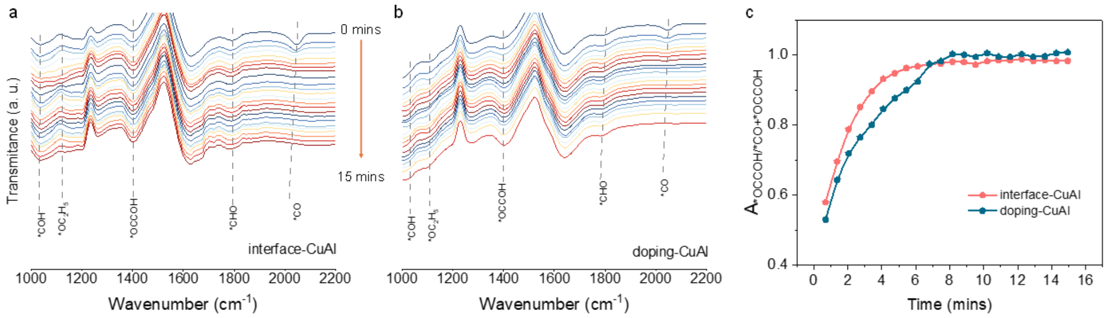

Figure S24. Time-independent intu-FTIR spectral analysis at -0.8 V vs. RHE for 15 mins on (a) interface-CuAl and (b) doping-CuAl. (c) $A_{*OCCOH\ /*OCCOH\ +*CO}$ ratio at interface-CuAl and doping-CuAl.

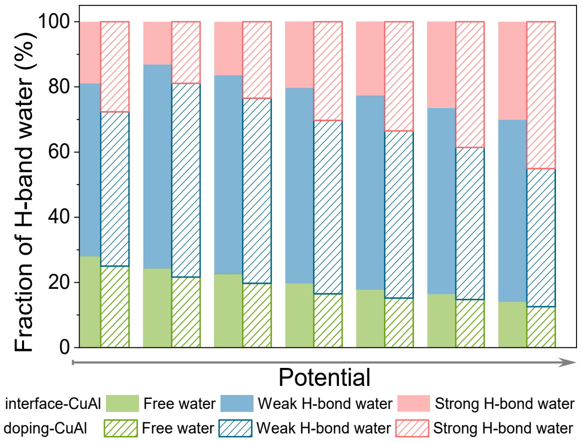

Figure S25. Variation in the proportion of interfacial water on interface-CuAl and doping-Cu.

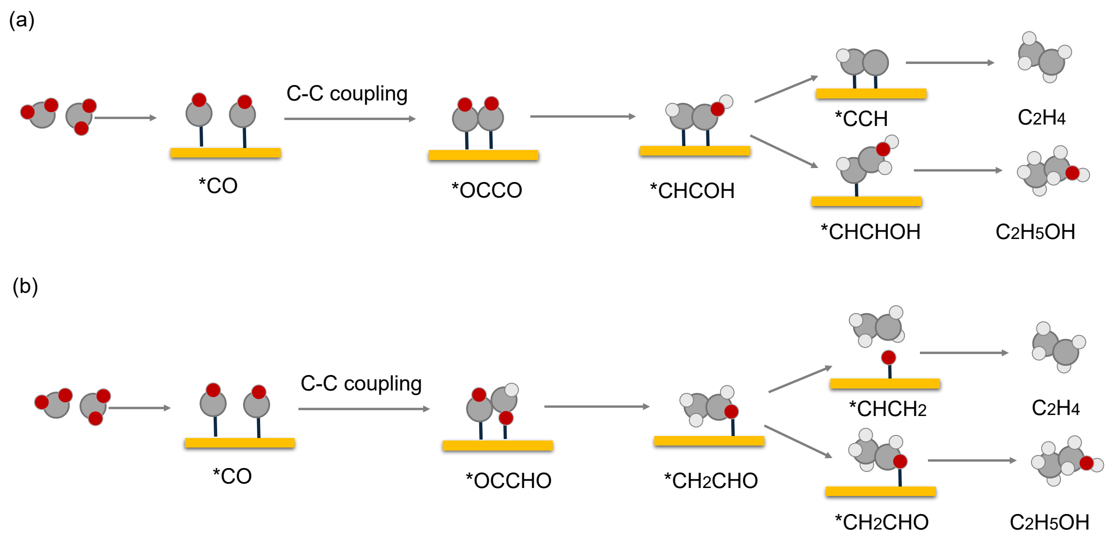

Figure S26. Proposed reaction pathway of CO_2RR to C_2H_4 and C_2H_5OH. (a)*OCCO pathway (b) *OCCHO pathway. Yellow: copper, gray: carbon, white: hydrogen, light red: oxygen.

The formation of C_2 products (ethylene and ethanol) during the CO_2 reduction reaction is commonly described by two competing pathways involving the *OCCO or *OCCHO intermediates[6], as summarized in Figure S26. In the *OCCO route, the key intermediate *CHCOH serves as a branching point: Path (i) proceeds via C–O bond scission, forming *CCH and eventually ethylene (C_2H_4); Path (ii) undergoes Cu–C bond cleavage followed by hydrogenation, yielding *CH_3CHO and ultimately ethanol (C_2H_5OH, Figure S26a). Alternatively, the *OCCHO pathway branches at the *CH_2CHO intermediate: C–O bond cleavage leads to ethylene, whereas C–O bond retention facilitates hydrogenation to *CH_3CHO, steering selectivity toward ethanol (Figure S26b). While techniques such as ATR-FTIR can offer insights into reactive intermediates, the low surface coverage and transient nature of species such as *CHCOH often prevent their direct detection. Therefore, mechanistic understanding in this work is built on the integration of in situ spectroscopic evidence and theoretical modeling. Our experimental and computational results consistently support *CHCOH as the dominant bifurcating intermediate under the studied conditions, leading us to propose the *OCCO pathway as the prevailing route.

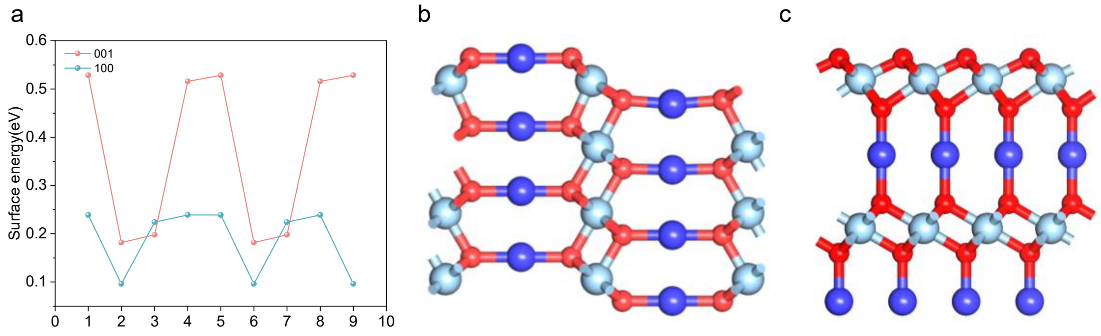

Figure S27. a The surface energies of $CuAlO_2(001)$ and $CuAlO_2(100)$, **b** The side view of $CuAlO_2(100)$, **c** The side view of $CuAlO_2(001)$.

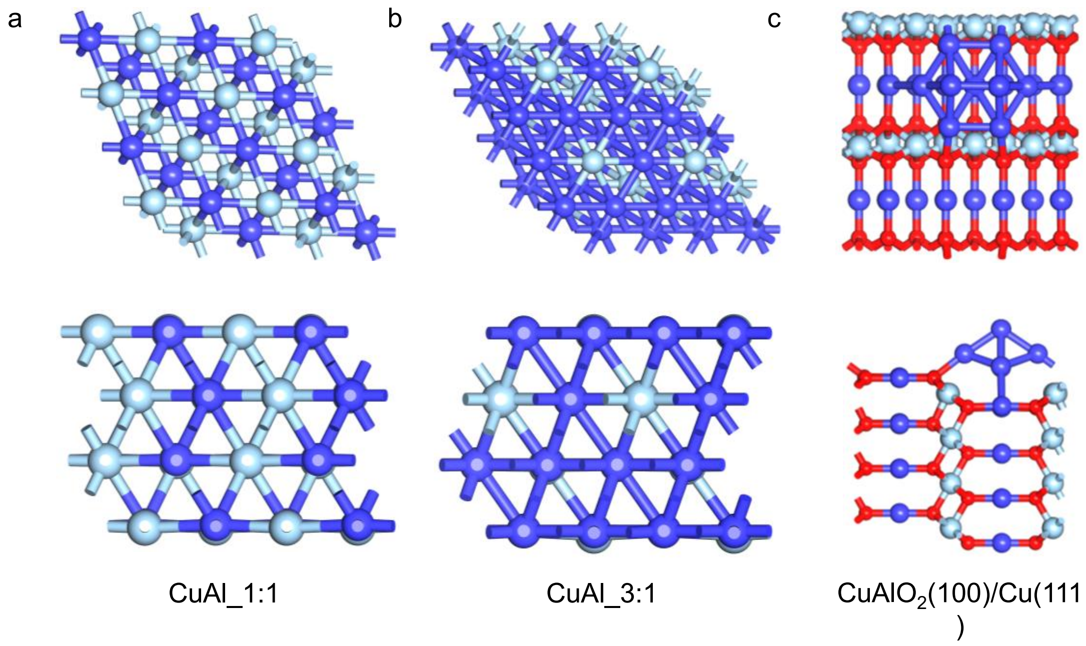

Figure S28. a The top and side view of CuAl_1:1. **b** The top and side view of CuAl_3:1, **c** The top and side view of $CuAlO_2$/Cu.

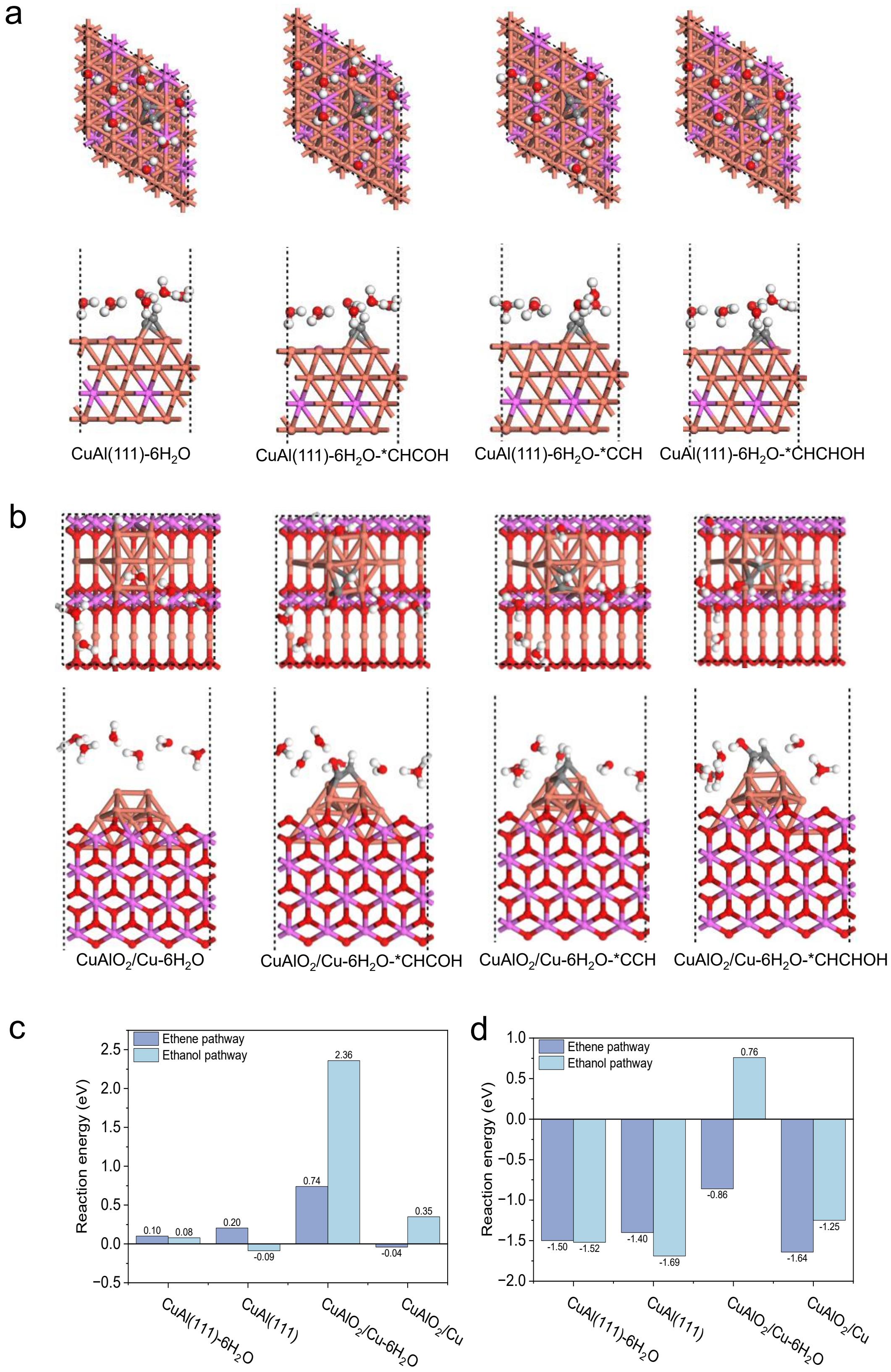

Figure S29 (a) Optimized structures of CuAl(111)-6H_2O with different reaction intermediates. From left to right: clean surface with 6 explicit water molecules, *CHCOH adsorbed intermediate, *CCH intermediate (ethylene pathway), and

*CHCHOH intermediate (ethanol pathway). Water molecules are included to represent the explicit solvent environment. (b) Optimized structures of $CuAlO_2$/Cu-6H_2O with different intermediates. From left to right: clean surface with 6 explicit water molecules, *CHCOH, *CCH (ethylene pathway), and *CHCHOH (ethanol pathway). The solvation effect is included by placing water molecules explicitly above the catalyst surface. Reaction free energies (ΔG) of ethylene and ethanol pathways on CuAl(111), CuAl(111)-6H_2O, $CuAlO_2$/Cu, and $CuAlO_2$/Cu-6H_2O at 0 V (c) and 0.7 V vs RHE (d).

In the explicit water solvent models CuAl(111)-6H_2O and $CuAlO_2$/Cu-6H_2O, it can be observed that despite changes in reaction energies compared to those without H_2O, the product selectivity of different catalysts remains unchanged: interface-$CuAlO_2$/Cu series catalysts prefer ethene production, while doping-CuAl series catalysts favor ethanol formation. Particularly in the interface-$CuAlO_2$/Cu-6H_2O system, the energy barrier for the ethanol pathway is significantly higher than for the ethene pathway, strongly suggesting that this catalyst surface favors ethene production. For doping-CuAl(111), we also tried adding 6H_2O and ultimately found that its main product remains unchanged as ethanol.

Figure S29d shows the energy comparison between ethylene and ethanol reaction pathways on different catalyst surfaces under 0.7 V vs RHE. The result indicate that even under different potential conditions, product selectivity remains consistent on most catalyst surfaces, demonstrating the stability and reliability of our calculation results. Particularly on the $CuAlO_2$/Cu-6H_2O surface, the energy difference between ethylene and ethanol pathways is most significant, further confirming our conclusions regarding catalyst selectivity.

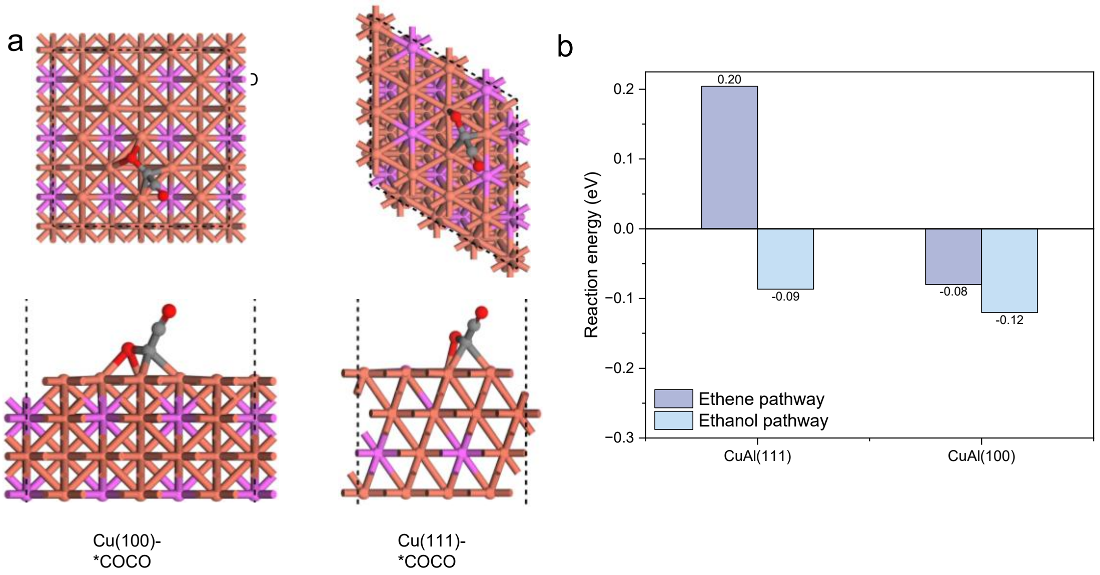

Figure S30. (a)Top and side views of the *COCOH intermediate adsorbed on CuAl(111) and CuAl(100) surfaces. Although CO–CO coupling is known to be challenging on (111) surfaces, our calculations confirm the feasibility of forming *COCOH on both facets. The corresponding reaction energies are 1.10 eV on CuAl(111) and 0.94 eV on CuAl(100). (b) Reaction energies for ethylene (*CHCOH → *CCH) and ethanol (*CHCOH → *CHCHOH) pathways on CuAl(111) and CuAl(100) surfaces. The consistent product selectivity across both surfaces supports the robustness of our model and indicates that facet variation does not alter the reaction outcome.

We have provided top and side views of the intermediates for this step on CuAl(111) and CuAl(100) (Figure S30a) to demonstrate that although this step is difficult to occur, it does indeed exist. Additionally, we calculated the reaction energies for this step on CuAl(111) and CuAl(100) surfaces, which are 1.10 eV and 0.94 eV respectively. Despite the relatively high-free energy change for this step, it can still occur from an energetic perspective.

we have performed additional calculations for the ethylene pathway (*CHCOH→*CCH) and ethanol pathway (*CHCOH→*CHCHOH) on the CuAl(100) surface as supplementary evidence (Figure S30b). The results indicate that the product selectivity remains consistent regardless of whether on the (111) or (100) crystal faces, further validating the reliability of our theoretical models.

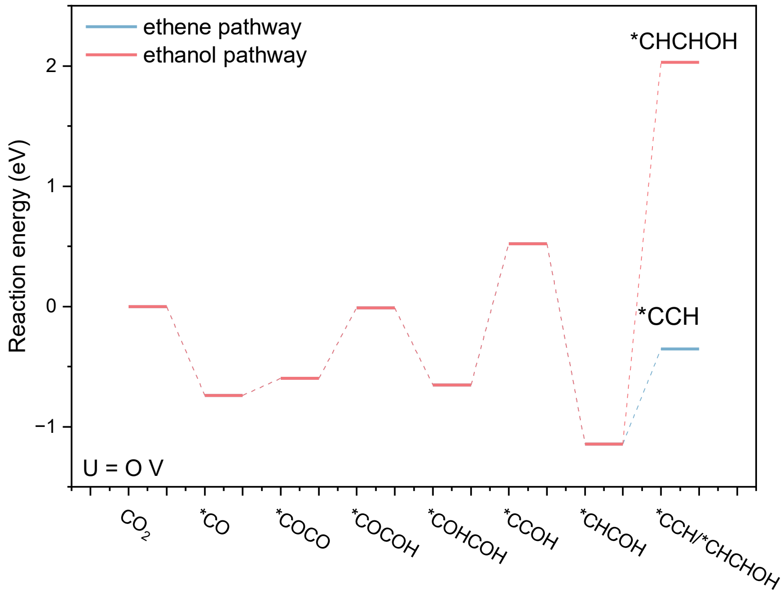

Figure S31. Calculated free energy profile for the CO_2 electroreduction pathway on the $CuAlO_2$/Cu-$6H_2O$ surface at U = 0 V vs RHE. Key intermediates include *CO, *COCO, *COCOH, *COHCOH, *CCOH, and *CHCOH, with the branching point leading to *CCH (ethylene) and *CHCHOH (ethanol).

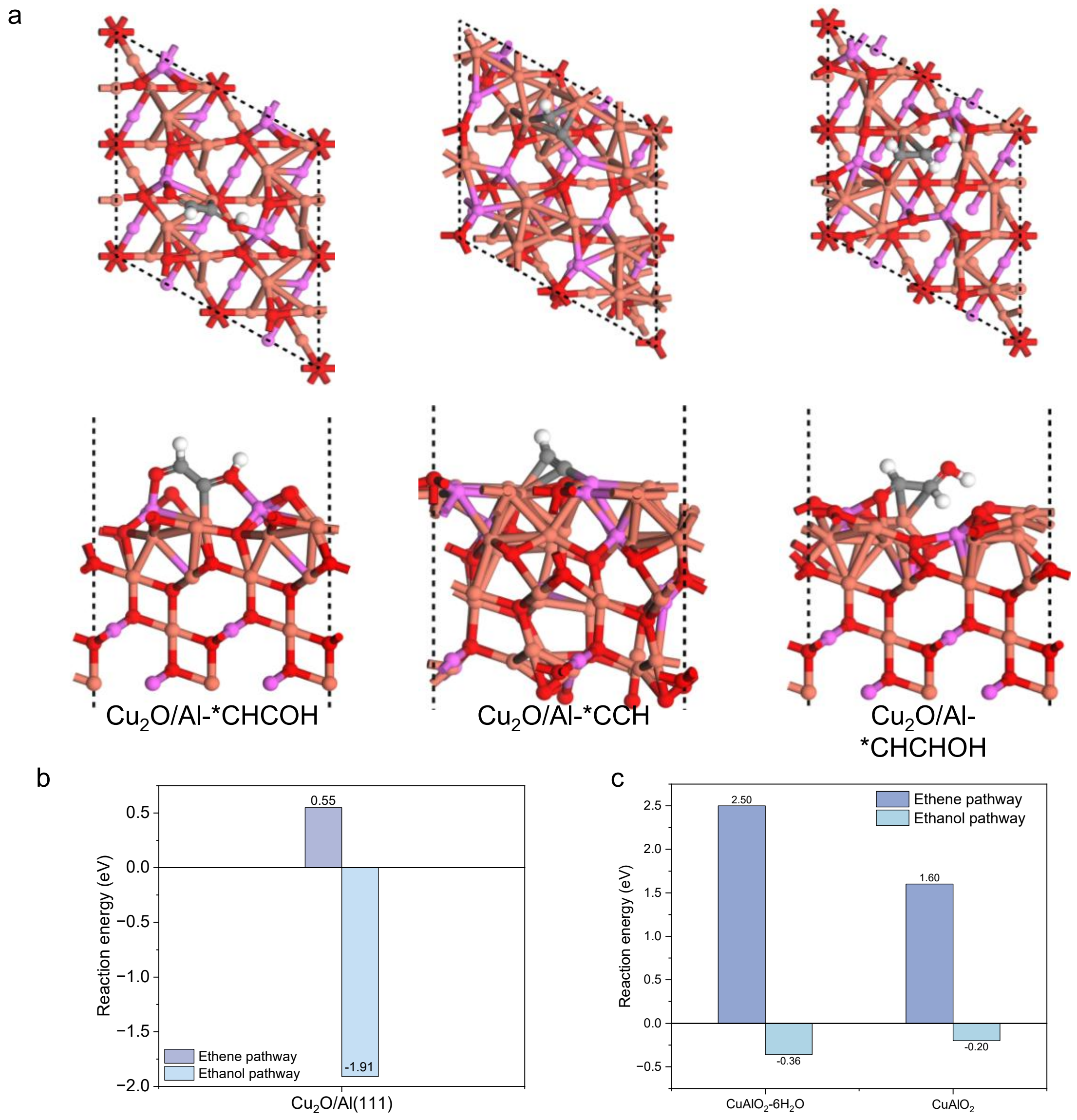

Figure S32. (a) The top and side views of *CHCOH, *CCH, *CHCHOH on Cu_2O/Al. (b) Comparison of reaction energies for ethylene and ethanol pathways on the Cu_2O/Al. (c) Comparison of reaction energies for ethylene and ethanol pathways on the $CuAlO_2$ region of the interface-CuAl, with and without explicit water molecules. Under no H_2O conditions, the ethylene pathway shows a higher reaction energy (1.60 eV) than the ethanol pathway (−0.20 eV). When explicit water molecules are introduced, the reaction energy for ethylene increases further to 2.50 eV, while that for ethanol becomes −0.36 eV.

As shown in Figure S32b, the reaction energy for the ethylene pathway is 0.55 eV, while that for the ethanol pathway is -1.40 eV. These results suggest that Cu_2O/Al intrinsically favors the formation of ethanol. Meanwhile, under no H_2O conditions, the reaction energy for the ethylene pathway is 1.60 eV, while that for the ethanol pathway is -0.20 eV. In contrast, under explicit water molecules conditions, the reaction energy for ethylene formation increases to 2.50 eV, whereas that for ethanol becomes -0.36 eV.

These results not only suggest an unrealistic reversal in selectivity—which contradicts experimental observations—but also indicate that the structures are unstable. These results suggest that $CuAlO_X$ intrinsically favors the formation of ethanol, and the observed shift in selectivity is likely driven by the synergistic interaction between $CuAlO_2$ and the Cu alloy interface.

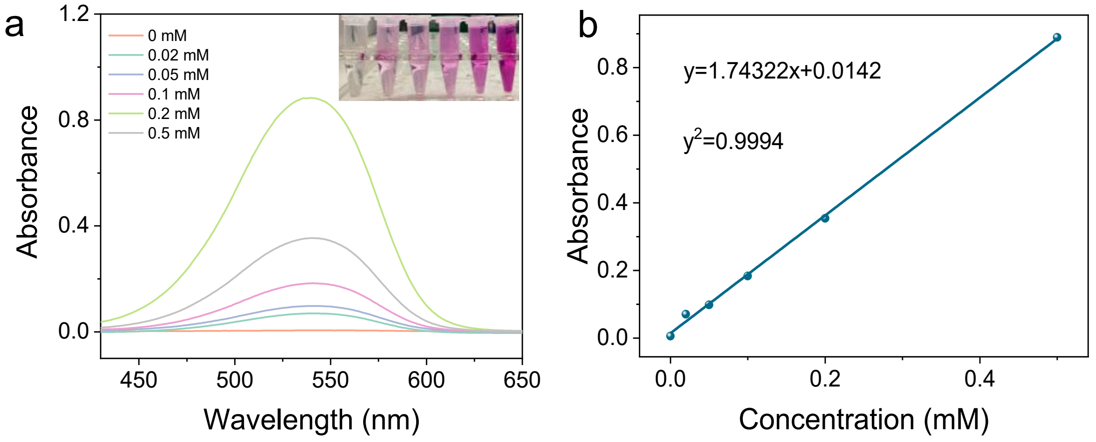

Figure S33. Calibration curves. UV-vis calibration curves of (a, b) nitrite (NO_2^-).

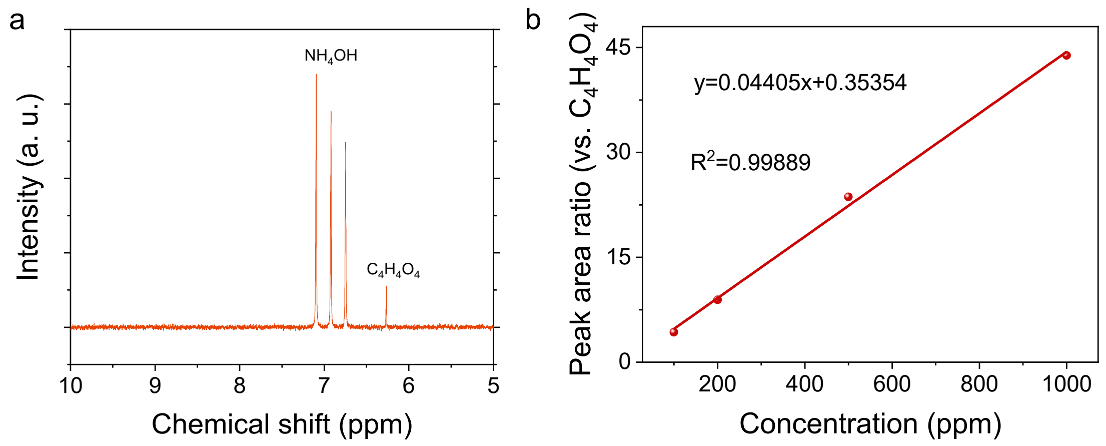

Figure S34. **Calibration curves.** (a) ^{1}H NMR calibration curve of NH_3 using different ammonium chloride concentration solutions of known concentration as standards. (b) ^{1}H NMR calibration curve of NH_3.

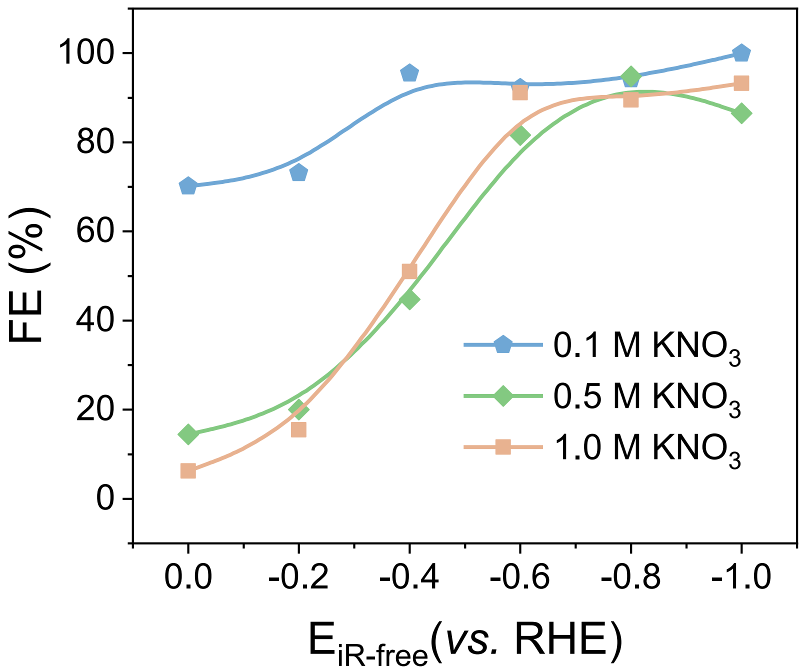

Figure S35. Electrochemical performances. Linear scan voltammetry curves of interface-CuAl in 1 M KOH + 0.1 M KNO_3, 1 M KOH + 0.5 M KNO_3 and 1 M KOH + 1 M KNO_3 with iR-80% compensation.

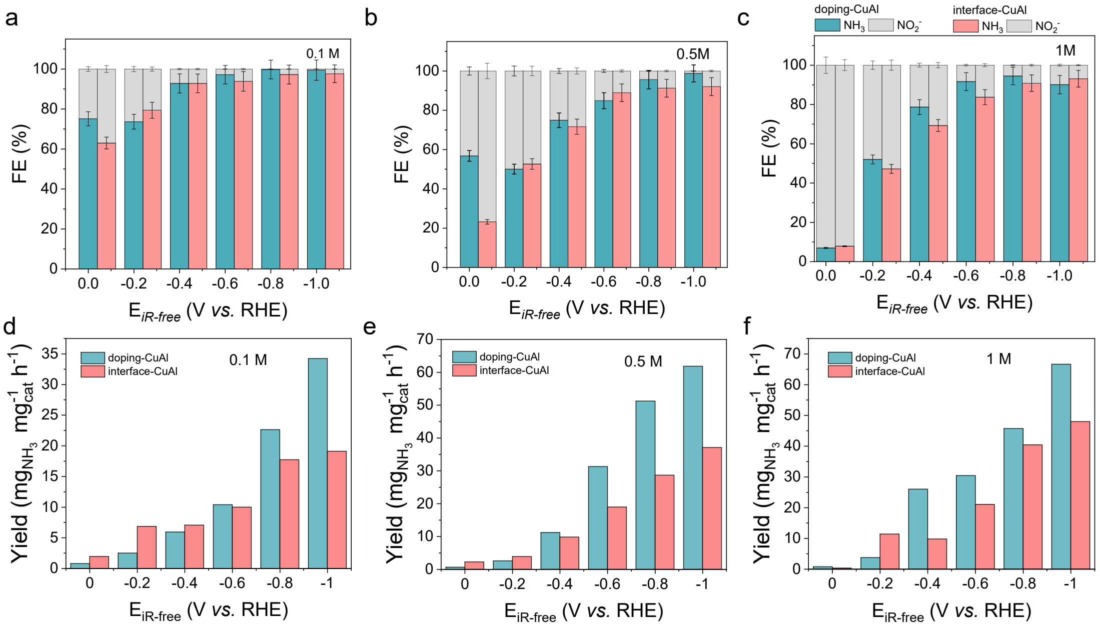

Figure S36. Electrochemical performances. **a-c**, FE of NH_3 and NO_2^- during NITRR of doping-CuAl and interface-CuAl in different KNO_3 concentrates (a) 0.1 M, (b) 0.5 M and (c) 1 M. **d-f**, Yield rate of NH_3 during NITRR of doping-CuAl and interface-CuAl in different KNO_3 concentrates (d) 0.1 M, (e) 0.5 M and (f) 1 M.

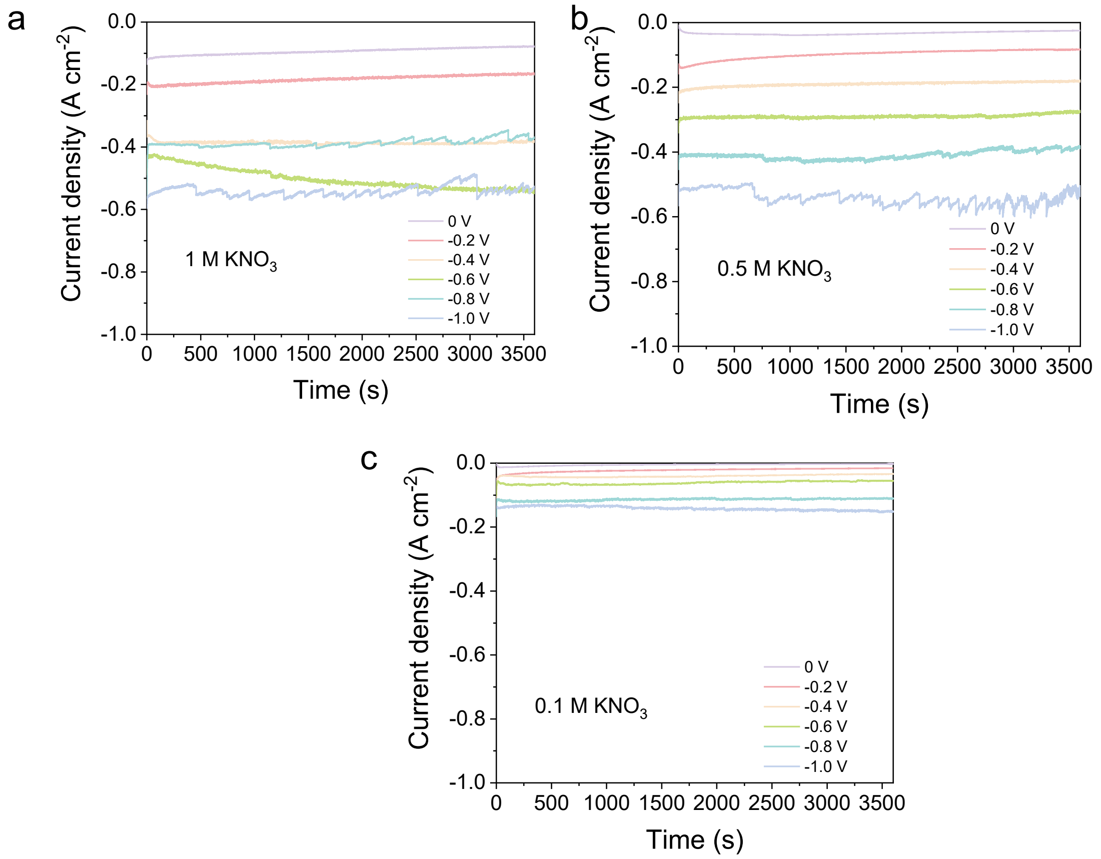

Figure S37. Electrochemical performances. Chronoamperometry of doping-CuAl in (a) 1 M KOH + 1 M KNO_3, (b) 1 M KOH + 0.5 M KNO_3 and (c) 1 M KOH + 0.1 M KNO_3 .

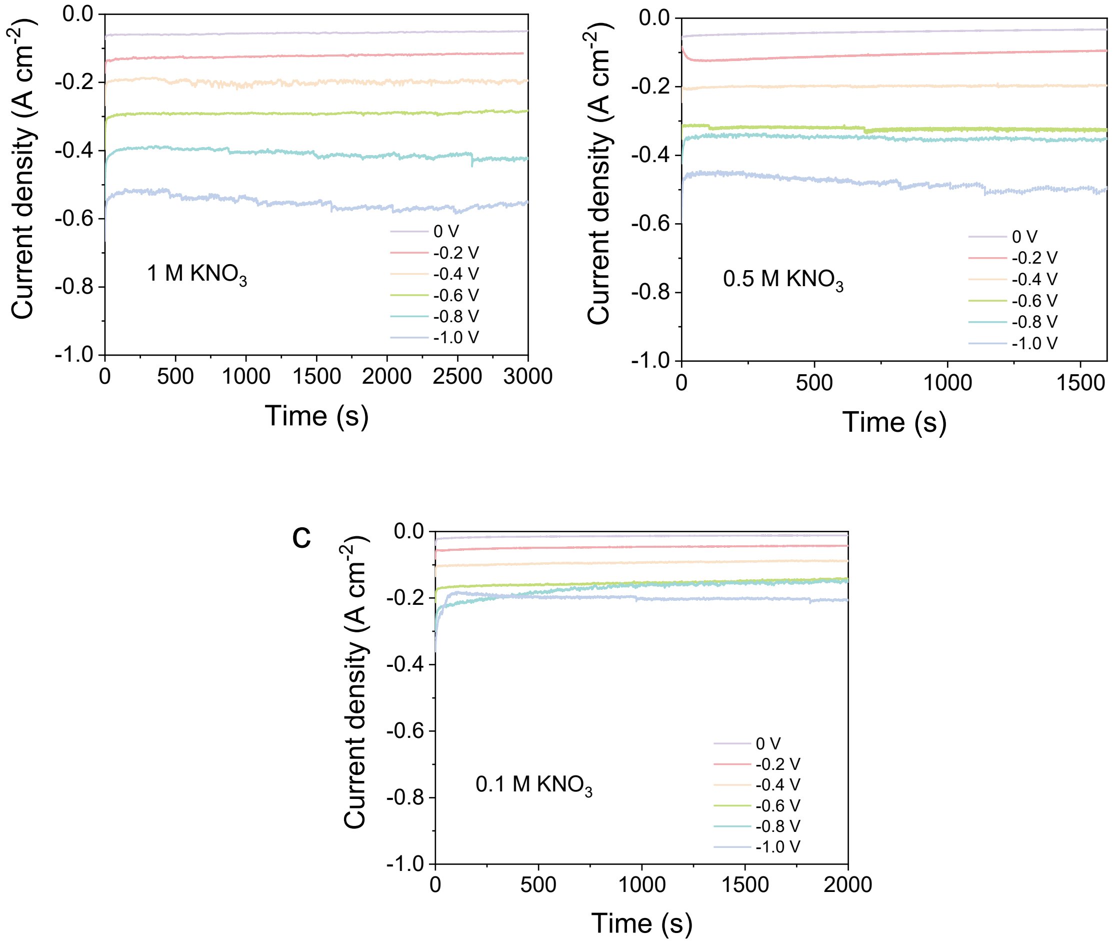

Figure S38. Electrochemical performances. Chronoamperometry of interface-CuAl in (a) 1 M KOH + 1 M KNO_3, (b) 1 M KOH + 0.5 M KNO_3 and (c) 1 M KOH + 0.1 M KNO_3.

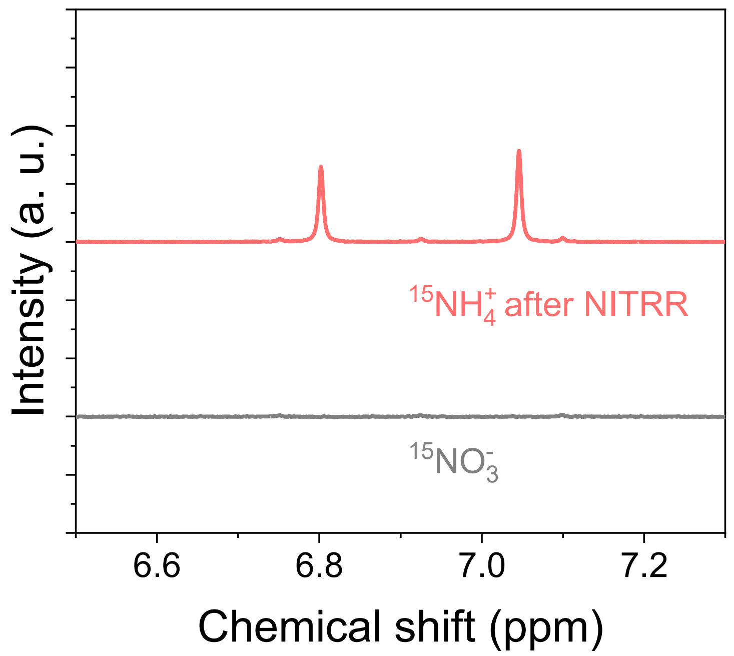

Figure S39. Product analysis. Representative ^{1}H-NMR spectrum NMR spectra before and after NITRR using $^{15}NO_3^-$ electrolytes.

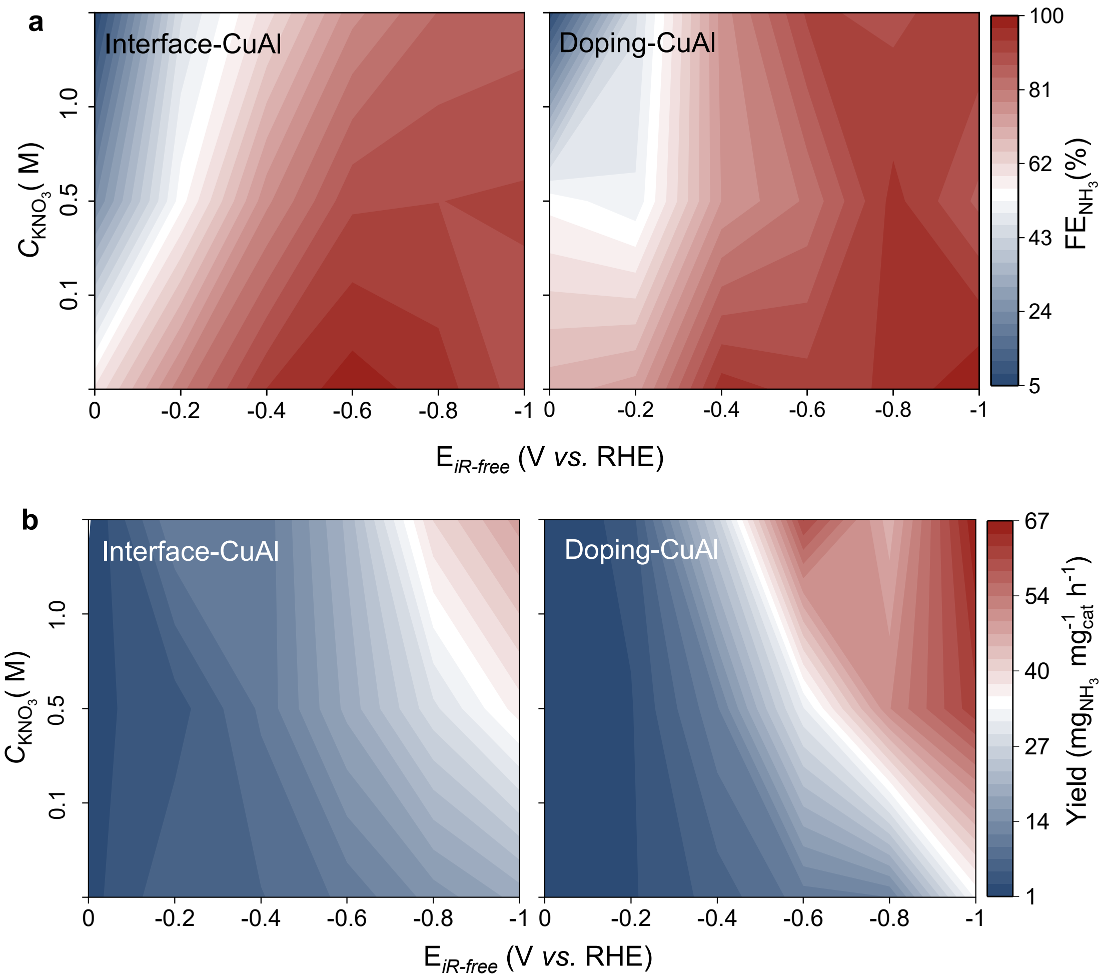

Figure S40. Electrocatalytic NITRR performance and mechanism. (**a, b**) 2D heatmaps of FE and Yield NH_3 in different electrolytes with different nitrate concentrations and potential in interface-CuAl and doping-CuAl.

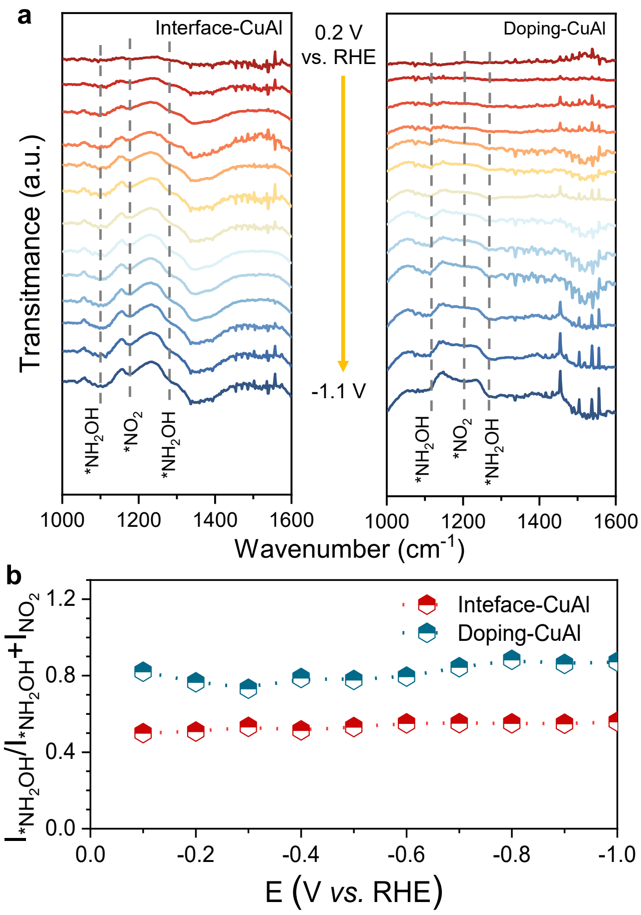

Figure S41. Electrocatalytic NITRR performance and mechanism. (a, b) In-situ ATR-FTIR measurement during NITRR and $I_{NO2OH}/I_{NO}+I_{NH2OH}$ in interface-CuAl and doping-CuAl.

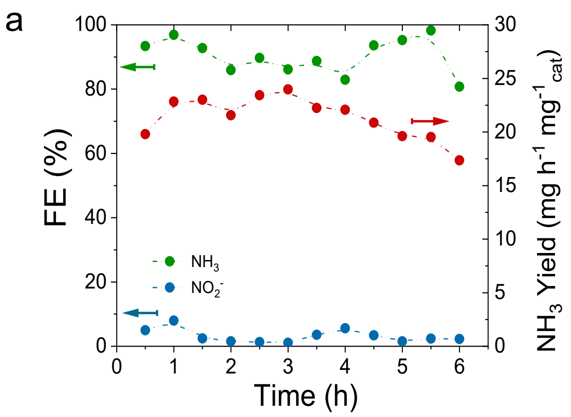

Figure S42. Electrochemical performances. Stability test for 6 h on (a) doping-CuAl catalyst and (b) interface-CuAl in 0.1 M KNO_3 on -0.8 V. (electrolyte refreshed every 0.5 hour).

For the stability test, we collected the electrolyte every half hour to measure the liquid products. Specifically, the electrolyte containing the liquid products was transferred to the sample collection tube by a pipette, and then the electrolytic cell was rinsed three times with the corresponding fresh electrolyte. After that, fresh electrolyte was added to the electrolytic cell for the next test. The stability test is carried out at room temperature and ambient pressure.

Table S1. Summary of ICP and EDX analysis results for Cu/Al ratio in interface-CuAl and doping-CuAl.

	ICP		EDX	
	Cu	Al	Cu	Al
interface-CuAl	68.9	31.1	67.1	32.9
doping-CuAl	90.3	9.7	93.6	6.4

Table S2. Summary of EDX analysis results for Cu/Al ratio in interface-CuAl and doping-CuAl after electrochemical testing.

	**Interface-CuAl**	**Doping-CuAl**
**Al**	16.41	2.12
**Cu**	83.59	97.89

Table S3. EXAFS fitting results of the Cu K-edge EXAFS for interface-CuAl and doping-CuAl, respectively.

Sample	path	CN	σ^2 (Å^2)	ΔE_0 (eV)	R (Å)	R-factor
Interface-Cu	Cu-O	4*	0.024(0.004)	0.09(0.02)	1.95(0.02)	0.007
	Cu-Cu1	1.2	0.082(0.009)	0.09(0.02)	2.94(0.03)	
	Cu-Cu2	1.2	0.007(0.005)	0.09(0.02)	2.78(0.03)	
Doping-Cu	Cu-O	4*	0.004(0.002)	1.02(0.13)	1.95(0.01)	0.018
	Cu-Cu	1.	0.006(0.004)	1.02(0.13)	2.97(0.09)	

S_0^2 is the amplitude reduction factor (0.9); CN is the coordination number; R is interatomic distance (the bond length between central atoms and surrounding coordination atoms); σ^2 is Debye-Waller factor (a measure of thermal and static disorder in absorber-scatterer distances); ΔE_0 is edge-energy shift (the difference between the zero kinetic energy value of the sample and that of the theoretical model). R-squared is used to value the goodness of the fit. *This value was fixed during EXAFS fitting, based on the known structure of CuO.

Table S4. The summary of FTIR peak assignments for the intermediate captured during CO_2RR.

**Band center (cm^{-1}) (This work)**	**Band center (cm^{-1}) (Literatures)**	**Intermediates**	**Refs.**
1035	~1050	*COH	*J. Am. Chem. Soc.* 144, 14936-14944, 2022. Ref.[7]
1130	~1150	*OC_2H_5	*Nat. Commun.* 13, 3754, 2022. Ref.[8]
1189<br>1179	~1200<br>~1415	*OCCOH	*Nat. Chem.* ***2025.*** Ref.[9]<br>*Nat. Commun* 16, 1956, 2025. Ref.[10]<br>*Angew. Chem. Int. Ed.* e202407612, 2024. Ref.[11]<br>*Nat. Commun.* 13, 3754, 2022. Ref.[8]
2000-2106	1885~2100	*CO	*Nat. Commun.* 15, 1257, 2024. Ref.[12]<br>*J. Am. Chem. Soc.* 146, 14260-14266, 2024. Rfe.[13]
3270	3270, 3250	4-HB water	*Nature Synthesis* 2025. Ref.[14]<br>*Nat. Commun.* 16, 2811, 2025. Ref[15]
3450	3450, 3450	2-HB water	
3600	3600, 3600	Free water	

Reference

[1] a) G. Kresse, J. Hafner, *Phys. Rev. B* **1993**, *47*, 558-561; b) G. Kresse, J. Hafner, *Phys. Rev. B* **1994**, *49*, 14251-14269; c) G. Kresse, J. Furthmüller, *Comput. Mater. Sci.* **1996**, *6*, 15-50.

[2] J. P. Perdew, K. Burke, M. Ernzerhof, *Phys. Rev. Lett.* **1996**, *77*, 3865-3868.

[3] P. E. Blöchl, *Phys. Rev. B* **1994**, *50*, 17953-17979.

[4] H. J. Monkhorst, J. D. Pack, *Phys. Rev. B* **1976**, *13*, 5188-5192.

[5] J. K. Nørskov, J. Rossmeisl, A. Logadottir, L. Lindqvist, J. R. Kitchin, T. Bligaard, H. Jónsson, *J. Phys. Chem. B* **2004**, *108*, 17886-17892.

[6] a) T. Cheng, H. Xiao, W. A. Goddard, 3rd, *Proc. Natl. Acad. Sci. U S A* **2017**, *114*, 1795-1800; b) H. Xiao, T. Cheng, W. A. Goddard, *J. Am. Chem. Soc.* **2016**, *139*, 130-136; c) F. Li, Y. C. Li, Z. Wang, J. Li, D.-H. Nam, Y. Lum, M. Luo, X. Wang, A. Ozden, S.-F. Hung, B. Chen, Y. Wang, J. Wicks, Y. Xu, Y. Li, C. M. Gabardo, C.-T. Dinh, Y. Wang, T.-T. Zhuang, D. Sinton, E. H. Sargent, *Nat. Catal.* **2020**, *3*, 75-82; d) J. Zhang, G. Zeng, S. Zhu, H. Tao, Y. Pan, W. Lai, J. Bao, C. Lian, D. Su, M. Shao, H. Huang, *Proc. Natl. Acad. Sci. U. S. A.* **2023**, *120*, e2218987120; e) Y. Liang, F. Li, R. K. Miao, S. Hu, W. Ni, S. Zhang, Y. Liu, Y. Bai, H. Wan, P. Ou, X.-Y. Li, N. Wang, S. Park, F. Li, J. Zeng, D. Sinton, E. H. Sargent, *Nat. Synth.* **2024**, *3*, 1104-1112.

[7] M. Zheng, P. Wang, X. Zhi, K. Yang, Y. Jiao, J. Duan, Y. Zheng, S. Z. Qiao, *J. Am. Chem. Soc.* **2022**, *144*, 14936-14944.

[8] P. Wang, H. Yang, C. Tang, Y. Wu, Y. Zheng, T. Cheng, K. Davey, X. Huang, S. Z. Qiao, *Nat. Commun.* **2022**, *13*, 3754.

[9] L. Li, C. Cui, *Nat. Chem.* **2025**.

[10] Y. Zhang, F. Chen, X. Yang, Y. Guo, X. Zhang, H. Dong, W. Wang, F. Lu, Z. Lu, H. Liu, H. Liu, Y. Xiao, Y. Cheng, *Nat. Commun.* **2025**, *16*, 1956.

[11] S. Li, G. Wu, J. Mao, A. Chen, X. Liu, J. Zeng, Y. Wei, J. Wang, H. Zhu, J. Xia, X. Wang, G. Li, Y. Song, X. Dong, W. Wei, W. Chen, *Angew. Chem. Int. Ed.* **2024**, e202407612.

[12] Y. Yao, T. Shi, W. Chen, J. Wu, Y. Fan, Y. Liu, L. Cao, Z. Chen, *Nat. Commun.* **2024**, *15*, 1257.

[13] Z. Liu, L. Song, X. Lv, M. Liu, Q. Wen, L. Qian, H. Wang, M. Wang, Q. Han, G. Zheng, *J. Am. Chem. Soc.* **2024**, *146*, 14260-14266.

[14] N. Ye, K. Wang, Y. Tan, Z. Qian, H. Guo, C. Shang, Z. Lin, Q. Huang, Y. Liu, L. Li, Y. Gu, Y. Han, C. Zhou, M. Luo, S. Guo, *Nat. Synth.* **2025**, *4*, 799–807.

[15] X. P. Yang, Z. Z. Wu, Y. C. Li, S. P. Sun, Y. C. Zhang, J. W. Duanmu, P. G. Lu, X. L. Zhang, F. Y. Gao, Y. Yang, Y. H. Wang, P. C. Yu, S. K. Li, M. R. Gao, *Nat. Commun.* **2025**, *16*, 2811.
